# Achieving Flexible Operation by Aggregating Structured Operating Protocols: An Electrolysis Case Study Using Real Market Data

**DOI:** 10.1021/acs.iecr.6c01628

**Published:** 2026-10-02

**Authors:** Jukbin Kim, Pen-Yeh Yen, Abiela M. Bradley, Rachel N. Gaines, Paul J. A. Kenis, Victor M. Zavala

**Affiliations:** † Department of Chemical and Biological Engineering, 5228 University of Wisconsin-Madison, Madison, Wisconsin 53706, United States; ‡ Department of Chemical and Biomolecular Engineering, 6146 University of Illinois Urbana−Champaign, Urbana, Illinois 61801, United States

## Abstract

Electrochemical devices can play a critical role in providing demand-side flexibility to the power grid, enabling greater integration of renewable power and enhancing system resilience. However, highly dynamic operation that tracks fluctuating market signals can accelerate degradation. To address this challenge, this work proposes a parallel aggregation framework in which multiple device modules operate under predefined structured protocols, defined as stable and periodic operating patterns assigned to individual units. Using a hydrogen electrolysis system participating in the real-time electricity market operated by the Electric Reliability Council of Texas (ERCOT) as a case study, we develop an optimization framework that determines optimal mixing of predefined structured protocols that minimizes total electricity costs. Our results show that the cost of the protocol-based parallel setting closely approximates that of fully flexible operation. This trend is consistently observed across multiple protocol sets and different market days, demonstrating the wide applicability of the proposed concept. The proposed approach can also enhance predictability and practical implementation.

## Graphical abstract

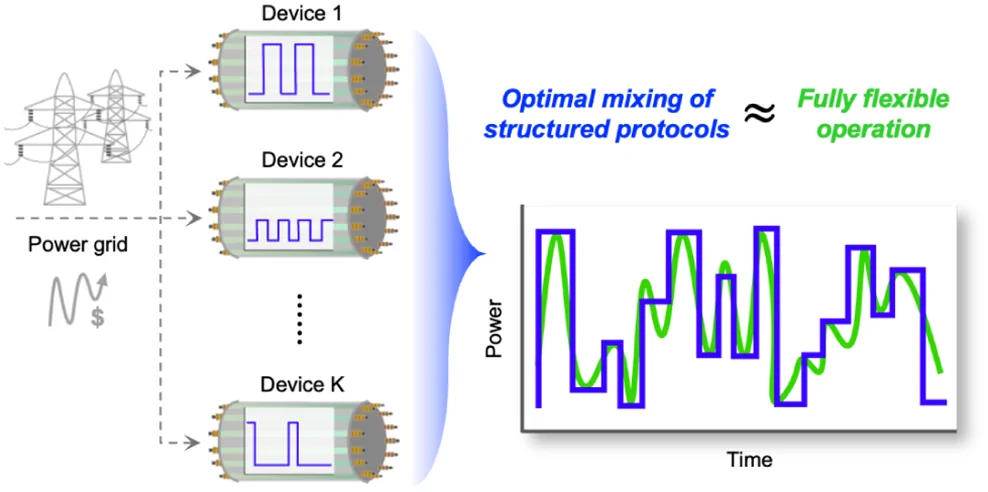

## 1 Introduction

The current energy landscape increasingly promotes the integration of renewable energy; however, managing the inherent volatility and intermittency of these resources remains a central challenge for power grid operators.^1^ For example, sudden drop in solar irradiance or fluctuations in wind speed can cause rapid changes in generation, creating real-time balancing challenges for grid operators.^2^

A promising approach for managing fluctuations is the utilization of grid-connected electrochemical devices.^3^ These devices can act as flexible controllable loads responding to electricity price variations and thereby shift loads to balance temporal and spatial supply demand mismatch. Such load flexibility can play a critical role in helping the grid absorb a larger amount of renewable power and in minimizing stranded generation.^4,5^ Specifically, hydrogen electrolysis systems can strategically schedule hydrogen production based on electricity price signals through demand-side participation in electricity markets. The produced hydrogen can then be supplied to end-use consumer or, alternatively, reconverted to electricity and sold back to the grid to generate revenue.^6^ Furthermore, the relatively low cost of storing and transporting hydrogen at large scale makes hydrogen electrolysis one of the most economically attractive forms of flexible electricity demand, allowing production to be shifted to periods of low electricity prices.^7^ In addition, electrochemical systems can participate in multiple electricity markets simultaneously, leveraging their different time resolutions to diversify transaction opportunities and enhance economic value.^8^

The U.S. power grid operates three major markets that electrolysis systems can exploit: the day-ahead market (DAM), the real-time market (RTM), and frequency regulation (FR).^9^ In the DAM, electricity is traded 1 day prior to physical delivery, while the RTM corrects deviations from the day-ahead schedule through real-time price clearing. FR is an ancillary service that adjusts power generation or consumption on the order of seconds to maintain system frequency. The nominal time resolutions of these markets range from hourly for the DAM, to 5 min or 15 min intervals for the RTM (depending on the region), and to subminute scales for FR.

Market participation of grid-connected devices offers benefits to both parties involved. While the power grid can be stabilized by grid balancing through electrochemical device participation,^10–12^ device operators can generate economic value by arbitraging electricity prices between peak and nonpeak periods.^13^ To respond effectively and strategically to electricity market signals, electrochemical devices are required to operate with a *high degree of dynamic flexibility*, allowing them to fully exploit fast price fluctuations and cut down costs. Such operational flexibility has been represented with different operating characteristics of devices, including dynamic load flexibility, ramp rates, and startup and shutdown times.^14–16^

Different studies have assessed flexibility for different electrolysis technologies and have proposed a range of operational scheduling strategies to enhance flexibility. Many of these approaches^17–20^ commonly allocate power across multiple independent stacks operating in parallel and apply differentiated control strategies to individual stacks. Some other studies have studied this problem from a computational viewpoint, by proposing advanced optimization formulations for handling complex dynamic operational decisions.^21,22^

Increasing dynamic flexibility often comes at the expense of reduced device durability.^23^ Specifically, highly flexible operation, which is characterized by frequent and rapid power adjustments, can accelerate degradation processes and shorten the lifetime of electrochemical systems.^24^ This trade-off has been characterized through laboratory experiments. For example, degradation rates of electrolysis cells have been examined under various operating schemes; results consistently report faster degradation under increased cycling intensity.^25,26^ These studies indicate that greater operational flexibility accelerates kinetically driven degradation mechanisms such as catalyst dissolution.

Despite evident trade-offs, few studies have examined device degradation in the context of operational flexibility. Most lab-scale studies focus on elucidating fundamental degradation mechanisms,^27^ while industrial-scale studies primarily assess the impact of degradation on process performance.^28–32^ However, relatively few studies have explored how operational strategies can be designed to enhance durability. Some studies have proposed switching strategies that reduce the number of start-up events to mitigate degradation;^33,34^ however, these do not address how to schedule operations driven by market dynamics.

To jointly address the operational flexibility and device durability, this work is motivated by the premise that operating electrolysis systems under structured schedules can help mitigate degradation. Building on this premise, we aim to understand how structured-schedule operation can maintain near-optimal economic performance while preserving a high degree of operational flexibility. Specifically, we propose a new operational paradigm that intentionally limits flexibility at the individual-device level by enforcing *structured protocol* which is a predefined operating profile that repeats with regular periodicity. By limiting rapid and arbitrary operational variations, this fixed protocol is expected to mitigate degradation or, at a minimum, make such degradation more predictable. This premise is motivated by experimental and simulation studies showing that degradation strongly depends on the operating profiles, with certain operating patterns exhibiting more favorable and predictable degradation behavior. For example, electrolyzer operation under different protocol types (hold, triangle-wave, square-wave) produces varying degrees of kinetic loss depending on ramping strategies and cycle frequencies.^25^ It has also been reported that the maximum degradation rate of an electrolyzer cell occurs at an intermediate power load (current density), rather than at the minimum or maximum power loads, and that prolonged operation at this operating point accelerates chemical degradation.^35,36^

Building on this foundation, our *working hypothesis* is that aggregating structured protocols of parallel devices in an optimal manner can achieve high system-level flexibility. This idea is motivated by prior studies which demonstrate that parallel operation of multiple units can substantially enhance overall flexibility (but those studies do not use structured protocols).^18,37^ More broadly, the proposed framework is conceptually inspired by the principle underlying Fourier analysis, in which complex and irregular signals can be approximated by the superposition of simpler constituent components.^38^ Analogously, we hypothesize that aggregating multiple simple, structured operating protocols can approximate the behavior of a highly flexible system.

Predefined scheduling and parallel operation are also well-established operating practices at the process scale, particularly in industrial settings that aim to have predictable behavior. Specifically, the requirement of capitalizing on volatile and unpredictable power market signals can complicate the practical implementation and performance forecasting of devices. Thus, industrial-scale operators generally prefer steady or structured operation with less uncertainty to ensure system stability and predictability.^39^ Moreover, most electrochemical systems are composed of multiple stacks,^40^ which are generally modular and independently controllable units.^20^

To test our working hypothesis, we propose a mathematical optimization framework that aims to optimally match operating protocols to market signals and with this minimizes total system cost. To benchmark the proposed strategy against conventional operational paradigms, we consider three electrolysis system configurations. Scenario (A) consists of a single electrolyzer operated in a fully flexible manner. Scenarios (B) and (C) both employ parallel operation of multiple electrolyzers, but differ in their operating strategies: scenario (B) maintains flexible operation for all units, whereas scenario (C) constrains each unit to predefined structured protocols. In Section 2, we detail the construction of each scenario and present their respective scheduling optimization formulations, all of which are modeled as mixed-integer linear programs (MILPs). To mathematically represent scenario (C), which is our proposed paradigm, we elaborate the necessity of defining structured protocols under a *new time scale* along with an additional operational strategy referred to as *operating mode*. In Section 3, we present case studies for a system participating in the RTM using historical data from the Electric Reliability Council of Texas (ERCOT). Our results indicate that the aggregation of structured protocols closely approximates the optimally flexible operation in terms of both operational trajectories and electricity cost. Similar trends are observed under different electricity market signals, suggesting that the proposed operating paradigm remains effective across varying market conditions. Our findings aim to motivate future experimental research that studies how degradation can be mitigated using structured operating protocols.

## 2 Methodology

### 2.1 Model Overview

To investigate the performance of the proposed *parallel operation of structured protocols*, this work constructed and compared three scenarios of grid-connected electrolysis operation, as shown in Figure 1. Scenario (A) shows the most basic operating scenario throughout the power grid to hydrogen demand, which utilizes a single electrolyzer. The electrolyzer purchases electricity from the power grid at the prevailing market price and converts it to produce hydrogen. We consider the electrolyzer system as an integrated process, in which auxiliary components, such as transformers, rectifiers, pumps, coolers, heaters, and compressors, are included in the total power requirements.^22^ The generated hydrogen is used immediately on demand or compressed and sent to storage for later usage or sale. The single electrolysis device considered here is assumed to operate in a flexible manner by sensibly and promptly capturing dynamic variations in electricity price. To regulate the degree of operational flexibility, the ramping behavior of electrolyzers serves as a principal control factor at grid-connected industrial scales;^41,42^ this aspect is discussed in detail in Section 2.2.1. Scenarios (B) and (C) operate multiple electrolyzers in parallel instead of running a single device and the aggregated operation of these units determines the total system behavior and hydrogen production. Scenario (B) allows all individual devices to operate flexibly under ramping constraints, as in scenario (A). Scenario (C) restricts each device to a predetermined structured protocol, and this new concept is elaborated in Section 2.2.3.

**Figure 1:**
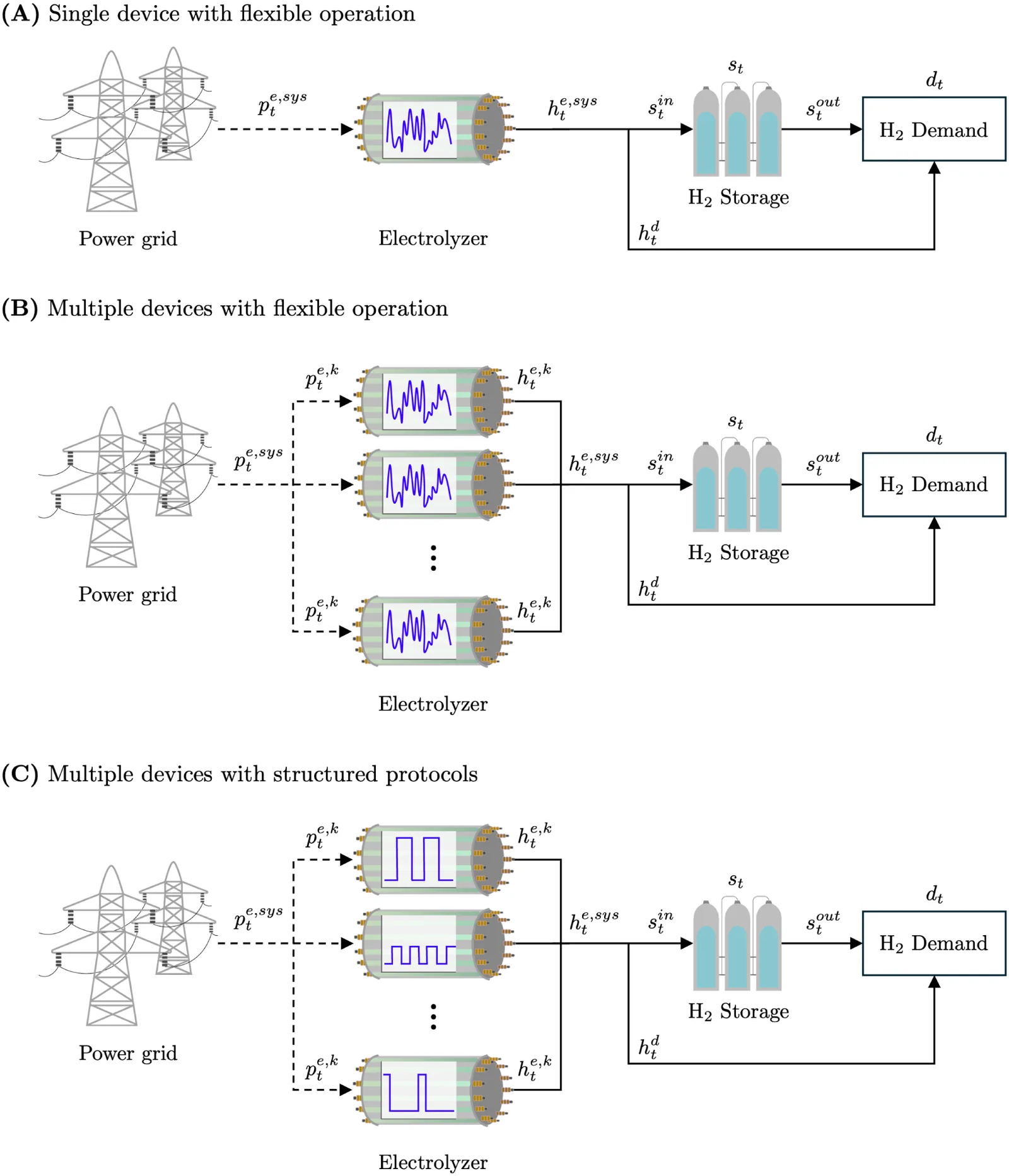
Schematics of grid-connected electrolysis systems under different operating scenarios, where dotted lines indicate power trajectories and solid lines represent hydrogen paths. (A) A single electrolyzer operating in a fully flexible manner. (B) Parallel operation of multiple electrolyzers, all operating flexibly. (C) Parallel operation of multiple electrolyzers operating under assigned structured protocols. Note that the aggregate electrolyzer capacity is identical across all scenarios to ensure a fair comparison.

### 2.2 Optimization Formulation

Since all scenarios share the same components except for the electrolyzer operating structure, we define a common set of indices, variables and parameters and formulate identical constraints to represent the shared characteristics across scenarios. Specifically, the power grid, hydrogen storage, and hydrogen demand are modeled using the same elements in all cases to ensure uniformity in the mathematical formulation. Beyond these aspects, the electrolysis system itself also exhibits several common operational properties across all frameworks. Each electrolyzer operates in one of two discrete states, ON or OFF. Its operation is characterized by both the state transitions and its operating behavior while in the ON state. In particular, a transition from the OFF state to the ON state incurs a start-up cost, corresponding to a cold start.^43^ Additionally, the power-to-hydrogen conversion of each electrolyzer is assumed to be linear, which is a commonly adopted approximation.^44^ This strategy is applicable across different electrolyzer technologies when experimental data are available for hydrogen production as a function of power consumption. Given these shared properties, we present the corresponding common mathematical formulations only in Section 2.2.1 to avoid repetition. The differences between the operating strategies are highlighted in Sections 2.2.2 and 2.2.3.

#### 2.2.1 Scenario (A)

As the basic operating scenario of grid-connected electrolysis system, scenario (A) adopts an optimization formulation that assumes a single electrolyzer. The optimization objective is to minimize the total electricity cost which consists of its operating electricity costs and start-up costs.

1 $$\underset{x_{A}}{\min}\underset{t\in T}{\sum }\left(\lambda_{t}^{e}p_{t}^{e,sys}+\lambda^{su}z_{t}^{su}\right)$$

$$x_{A}=\left\{p_{t}^{e,sys},h_{t}^{e,sys},h_{t}^{d},s_{t},s_{t}^{in},s_{t}^{out},d_{t},z_{t}^{on},z_{t}^{off},z_{t}^{su}\right\}$$

The electricity price $\lambda_{t}^{e}$ represents the given market data, such as real-time or day-ahead price, and is multiplied by the power consumption of electrolyzer $p_{t}^{e,sys}$ to compute the operating electricity cost. The time index t∈T corresponds to the temporal resolution of the market data supplied to the system (e.g., RTM or DAM). For example, when DAM data with hourly resolution are used, each time step *t* represents 1 h as the operating decisions for all devices can be updated at hourly intervals. Along with operating cost, start-up cost $\lambda_{t}^{su}$ is added to the operating cost during start-up period, as indicated by the binary start-up variable $z_{t}^{su}$ .

Electrolyzer operation at each time *t* is enforced by the following set of constraints:

2 $$1=z_{t}^{on}+z_{t}^{off},,\forall t\in T$$

3a $$z_{t}^{su}\geq z_{t}^{on}-z_{t-1}^{on},,\forall t\in T\backslash \left\{1\right\}$$

3b $$z_{t=1}^{su}=0$$

4a $$p_{t}^{e,sys}\leq P^{max}z_{t}^{on},,\forall t\in T$$

4b $$p_{t}^{e,sys}\geq P^{min}z_{t}^{on},,\forall t\in T$$

5 $$\left|p_{t}^{e,sys}-p_{t-1}^{e,sys}\right|\leq P^{max}R,,\forall t\in T\backslash \left\{1\right\}$$

6 $$h_{t}^{e,sys}=Ap_{t}^{e,sys}+Bz_{t}^{on},,\forall t\in T$$

Constraint ([Disp-formula eq2]) ensures that the electrolyzer operates at two mutually exclusive states, ON or OFF, and constraints ([Disp-formula eq3]) and ([Disp-formula eq4]) define the restrictions for a cold start-up. Under its ON state, the power consumption of a single electrolyzer is constrained by its maximum load *P* ^*max*^ ([Disp-formula eq5]), minimum load *P* ^*min*^ ([Disp-formula eq6]), and ramping ratio *R* ([Disp-formula eq7]). The ramping ratio *R* is a value between $\frac{P^{min}}{P^{max}}\leq R\leq 1$ that limits the change of power consumption by time. If $R<\frac{P^{min}}{P^{max}}$ , the ramping limit is too restrictive to move from *P* ^*min*^ to 0 in one time step, which prevents shutdown. Under the given range of *R*, a larger value allows for greater changes in power consumption within a single time step, enabling the electrolyzer to operate under highly fluctuating conditions. In contrast, a smaller value of *R* tends to stabilize the electrolyzer operation by restricting the rate of change in power consumption. Thus, ramping tuning is a typical mechanism to control the operational flexibility. The constraint ([Disp-formula eq8]) represents a linear hydrogen production curve characterized by electrolyzer-specific parameters *A* and *B*.

Additional constraints regarding hydrogen storage and demand are

7a $$h_{t}^{e,sys}=h_{t}^{d}+s_{t}^{in},,\forall t\in T$$

7b $$d_{t}=h_{t}^{d}+s_{t}^{out},,\forall t\in T$$

7c $$s_{t}=s_{t-1}+s_{t}^{in}\mu -s_{t}^{out}\mu ,,\forall t\in T\backslash \left\{1\right\}$$

7d $$s_{t=1}=S^{ini}+s_{t=1}^{in}\mu -s_{t=1}^{out}\mu$$

7e $$s_{t}\leq C^{S},,\forall t\in T$$

7f $$s_{t}^{out}\leq C^{S,out},,\forall t\in T$$

7g $$\underset{t\in T_{i}^{d}}{\sum }d_{t}\mu \geq D_{i}^{min},,\forall i\in I$$

Constraints ([Disp-formula eq9]) and ([Disp-formula eq10]) capture the hydrogen balance that governs its production, storage, and demand. The dynamics balance in storage is in particular described by constraints ([Disp-formula eq11]) and ([Disp-formula eq12]) where the storage capacity *s*_*t*_ and its outflow $s_{t}^{out}$ are controlled by constraints ([Disp-formula eq13]) and ([Disp-formula eq14]), respectively. We note that the unit of storage *s*_*t*_ is kg, while the units of flow rates $s_{t}^{in}$ and $s_{t}^{out}$ are kg/h; this is why the flow rates are multiplied by the length of electricity market time resolution μ. For example, μ = 1 h for the hourly day-ahead market and μ = 0.25 h for the 15 min real-time market. Lastly, the total hydrogen demand over a subperiod of constraint ([Disp-formula eq15]) should satisfy its minimum value where the subperiod may represent several hours, a day, or similar time frames.

#### 2.2.2 Scenario (B)

Scenario (B) is an extension of scenario (A) in which parallel devices are used instead of a single device. To identify individual devices in the mixture of all operating units, we need a new index k∈K where K stands for the set of all devices, as well as a new set of variables indicating the power consumption $p_{t}^{e,k}$ , hydrogen production $h_{t}^{e,k}$ , and operational states $z_{t}^{on,k}$ , $z_{t}^{off,k}$ , $z_{t}^{su,k}$ of each electrolyzer *k*. With these new device-specific variables, the start-up cost term of objective ([Disp-formula eq1]) is transformed into the second term in ([Disp-formula eq16]) that sums the start-up costs of all eletrolyzers inside the system.

8 $$\underset{x_{B}}{\min}\underset{t\in T}{\sum }\lambda_{t}^{e}p_{t}^{e,sys}+\underset{k\in K}{\sum }\underset{t\in T}{\sum }\lambda^{su,k}z_{t}^{su,k}$$

$$x_{B}=\left\{p_{t}^{e,k},p_{t}^{e,sys},h_{t}^{e,k},h_{t}^{e,sys},h_{t}^{d},s_{t},s_{t}^{in},s_{t}^{out},d_{t},z_{t}^{on,k},z_{t}^{off,k},z_{t}^{su,k}\right\}$$

Similarly, the electrolyzer operation-related constraints ([Disp-formula eq2]–[Disp-formula eq8]) are generalized to ([Disp-formula eq17]–[Disp-formula eq23]) with superscript *k* and the corresponding device-specific parameters *P* ^*max*,*k*^, *P* ^*min*,*k*^, *R* ^*k*^, *A* ^*k*^, and *B* ^*k*^. Also, additional constraints ([Disp-formula eq24]) and ([Disp-formula eq25]) are required to integrate power consumption and hydrogen production across all electrolyzers, thereby defining the total system variables $p_{t}^{e,sys}$ and $h_{t}^{e,sys}$ . These two variables are the same with those used in scenario (A) to represent the power consumption and hydrogen production of a single device, which indicates that scenario (A) is a special case of scenario (B) with *k* = 1. As explained above, scenario (B) still takes advantage of constraints ([Disp-formula eq9]–[Disp-formula eq15]) to describe the hydrogen storage and demand limitations.

9 $$1=z_{t}^{on,k}+z_{t}^{off,k},,\forall t\in T,\forall k\in K$$

10a $$z_{t}^{su,k}\geq z_{t}^{on,k}-z_{t-1}^{on,k},,\forall t\in T\backslash \left\{1\right\},\forall k\in K$$

10b $$z_{t=1}^{su,k}=0,,\forall k\in K$$

11a $$p_{t}^{e,k}\leq P^{max,k}z_{t}^{on,k},,\forall t\in T,\forall k\in K$$

11b $$p_{t}^{e,k}\geq P^{min,k}z_{t}^{on,k},,\forall t\in T,\forall k\in K$$

12 $$\left|p_{t}^{e,k}-p_{t-1}^{e,k}\right|\leq P^{max,k}R^{k},,\forall t\in T\backslash \left\{1\right\},\forall k\in K$$

13 $$h_{t}^{e,k}=A^{k}p_{t}^{e,k}+B^{k}z_{t}^{on,k},,\forall t\in T,\forall k\in K$$

14 $$p_{t}^{e,sys}=\underset{k\in K}{\sum }p_{t}^{e,k},,\forall t\in T$$

15 $$h_{t}^{e,sys}=\underset{k\in K}{\sum }h_{t}^{e,k},,\forall t\in T$$

#### 2.2.3 Scenario (C)

The operational flexibility in the conventional scenarios (A) and (B) is predominantly dictated by the ramping ratio *R* (or *R* ^*k*^), with the highest degree of flexibility attained at R = 1. However, the inherent trade-off between flexibility and durability implies that highly flexible operation can accelerate electrolyzer degradation, thereby increasing replacement frequency and elevating the associated capital costs. In addition, pronounced fluctuations in operation are generally unfavorable in practical process environments due to their limited feasibility and reduced predictability. Thus, this work proposes an operating strategy based on predefined, simple, and periodic protocols to overcome the flexibility-durability trade-off and enhance operational feasibility and predictability. They are expected to mitigate electrolyzer degradation compared to highly volatile operation. In addition, this approach improves practical implementability and has already been adopted in real-world systems, offering enhanced predictability in both performance and device lifespan.

From a flexibility viewpoint, relying on predefined protocols for a device partially limits its ability to respond to electricity market variations. To increase flexibility, the present work uses *parallel operation* of multiple electrolyzers where each is assigned a distinct *structured protocol*. This approach is motivated by a hypothesis that combining diverse structured protocols can yield a high degree of operational flexibility, even though any single predefined protocol is inherently limited in its flexibility. This concept is also consistent with common practices in electrochemical systems, which are typically designed as modular and independently controlled units. If the aggregate operation of structured protocols can approximate ideally (fully) flexible operation corresponding to R = 1 in conventional scenarios, as illustrated in Figure 2, then this operating paradigm has the potential to deliver high flexibility while simultaneously mitigating degradation and circumventing other practical challenges. To clarify the scope of this work, degradation effects associated with structured protocols are not explicitly modeled; instead, this work is devoted to demonstrating the proposed hypothesis through case studies using real grid data.

**Figure 2:**
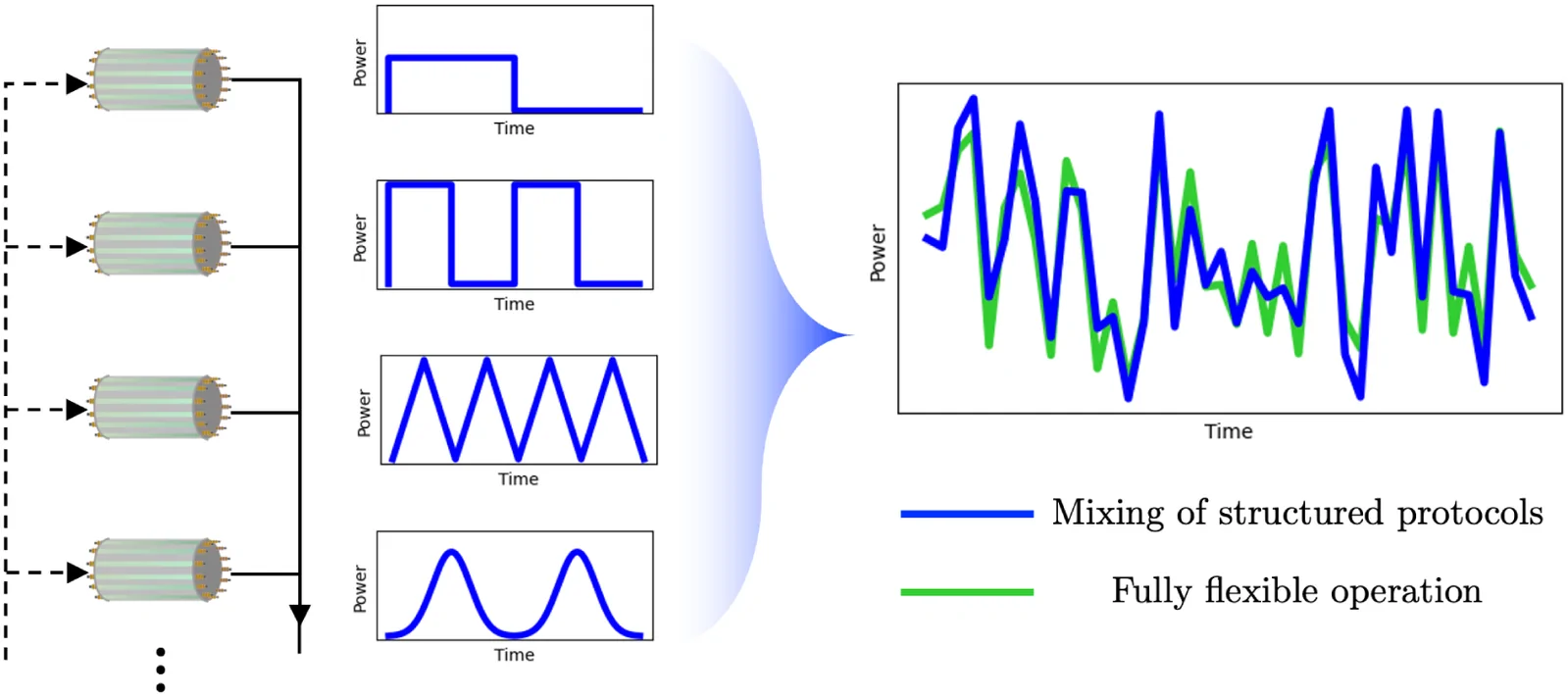
Conceptual sketches illustrating the core hypothesis of this study: appropriate mixing of structured protocols can approximate fully flexible operation.

##### 2.2.3.1 Allocation of Structured Protocols

We define a structured protocol as an operational basis which (i) specifies the operating behavior of a device over a designated time interval, which we name as *time slot σ*, and (ii) can repeat identically across successive slots without internal modification. Structured protocols can have any types of waveform, such as square waves, triangular waves, and Gaussian-shape waves, as depicted in Figure 2. Here we focus on square-wave protocols for simplicity, as other waveform types can be generated through transformations of square-wave bases. For example, whereas square waves induce abrupt changes in power consumption, triangular waves vary more gradually; thus, triangular patterns can be constructed by appropriately ramping square waves. Similarly, Gaussian-shaped profiles can be approximated by discretizing the time domain and combining multiple triangular waves with diverse ramp rates. Consequently, square waves provide a suitable foundation for exploring the proposed operating paradigm.

Each device needs to be assigned a specific time-slot length and a fixed protocol within the time slot. We denote the set of entire operating time steps T={t|t=1,2,...,T} and designate a particular length of time slot *N*_*k*_ to an electrolyzer *k*. Then the number of time slot *S*_*k*_ is an integer that rounds up *T*/*N*_*k*_ and each time slot σ becomes an element of a set $S_{k}=\left\{\sigma |\sigma =1,\;2,\;...,\;S_{k}\right\}$ . The time slot σ hereby becomes a time domain over which electrolyzer scheduling decisions are made, or the time resolution. Whereas device operation in conventional scenarios (A) and (B) is determined at every refined time step *t*, scenario (C) replaces this with a time slot σ making it a coarser time resolution for device scheduling.

Additionally, to distinguish from the original time step *t*, let us introduce an inner-slot time step τ. Then the set $N_{k}=\left\{\tau |\tau =1,\;2,\;...,\;N_{k}\right\}$ becomes a temporal domain where to define a protocol for an electrolyzer *k*. Although τ has an identical time interval *t*, introducing the additional time scale τ is necessary for presetting protocols within a single time slot in a manner that is distinct from the original time domain. Using these new temporal indices and sets, we define a two-dimensional time representation (σ, τ), which denotes the τ-th subtime step within the σ-th time slot. It has one-to-one correspondence with the existing *t*-time scale via a relationship *t* = *N*_*k*_ × (σ – 1) + τ.

For a square-wave protocol where no ramping occurs, the protocol profile can be represented as a set of constant parameters $P_{\tau }^{prot,k}$ that specify the fixed power load of electrolyzer k∈K at each inner-slot time step $\tau \in N_{k}$ . Each parameter takes a value within the prescribed minimum and maximum power limits of electrolyzer *k*, ensuring that the device remains online within a single time slot and thereby avoids intraslot start-up costs. For simplicity, any square-wave protocol assigned to device *k*, which is the collection of $P_{\tau }^{prot,k}$ over $\tau \in N_{k}$ , is assumed to alternate periodically between the minimum power level *P* ^*min*,*k*^ and a designated operating power level specific to the protocol. Under this representation, each square-wave protocol can be fully characterized by three parameters: power load, period length, and duty cycle. The power load marks the amplitude of the periodic profile, the period length specifies the duration of one complete cycle within a time slot, and the duty cycle denotes the fraction of the period during which the electrolyzer operates at the designated power load rather than *P* ^*min*,*k*^. Table 3 and Figure 6 provide a numerical and visual illustration of the protocol characterization in terms of the slot length and the three defining parameters.

##### 2.2.3.2 Optimal Scheduling with Operating Modes

Under the proposed protocol-based framework, a primary operational strategy is that, at each time slot $\sigma \in S_{k}$ , any device k∈K can select one of three operating options: operating according to its assigned protocol, operating at its maximum power level *P* ^*max*,*k*^, or operating at its minimum power level *P* ^*min*,*k*^, as illustrated in Figure 3. The purpose of this scheme is to relax otherwise rigid operational constraints and provide the system with a minimal yet meaningful degree of flexibility. If every device were strictly constrained to follow its predefined protocol over the entire operating horizon without any adjustments, the aggregated system operation would exhibit nearly identical behavior over time and thus be unable to leverage the electricity market variations. To avoid this limitation, we allow each electrolyzer *k* to select its *operating mode* at every time slot σ, choosing between the assigned protocol and one of the extreme power levels. This mode selection is permitted to vary across time slots in response to time-varying electricity market signals. Through this mechanism, the system can access a portion of the flexibility benefits of dynamic operation, even though the resulting profiles do not achieve the full flexibility attainable under an optimally flexible control policy.

**Figure 3:**
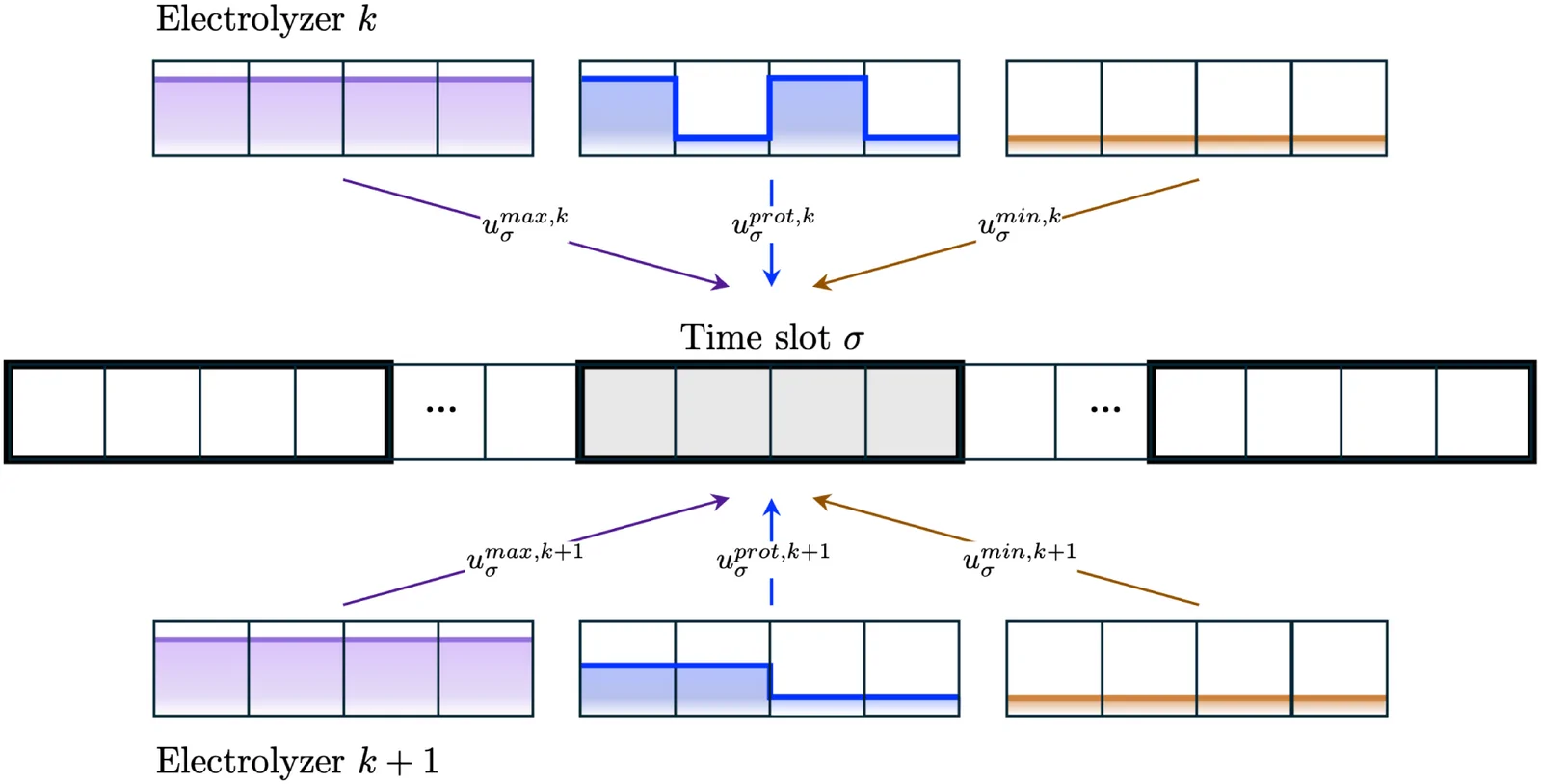
Conceptual sketch describing the operating mode selection for electrolyzer *k* at time slot σ.

From an optimization perspective, the proposed framework can be interpreted as a *dynamic assignment problem* of different operating modes, since exactly one operating mode must be selected at each scheduling interval. Unlike scenarios (A) and (B), scenario (C) introduces a new set of electrolyzer decision variables over the two-dimensional temporal domain (σ, τ). Because the time slot σ serves as the scheduling resolution, the binary variables representing ON, OFF, and start-up states in the original *t*-scale $\left\{z_{t}^{on,k},\;z_{t}^{off,k},\;z_{t}^{su,k}\right\}$ are replaced by their counterparts in the σ-domain $\left\{u_{\sigma }^{on,k},\;u_{\sigma }^{off,k},\;u_{\sigma }^{su,k}\right\}$ . In addition, we introduce binary variables $u_{\sigma }^{prot,k}$ , $u_{\sigma }^{max,k}$ , and $u_{\sigma }^{min,k}$ , which correspond to the three mutually exclusive operating modes. These variables respectively indicate whether electrolyzer *k* operates according to its assigned protocol, or continuously at its maximum or minimum power load within time slot σ. Lastly, continuous variables $p_{\sigma ,\tau }^{e,k}$ and $h_{\sigma ,\tau }^{e,k}$ are defined over (σ, τ)-scale to compute the power consumption and hydrogen production of electrolyzer *k* at the inner-slot resolution.

The objective ([Disp-formula eq26]) follows a formulation similar to that of ([Disp-formula eq16]) for scenario (B), with the key distinction that the start-up costs are applied at the time-slot level σ rather than at the time-step level *t*.

16 $$\underset{x_{C}}{\min}\underset{t\in T}{\sum }\lambda_{t}^{e}p_{t}^{e,sys}+\underset{k\in K}{\sum }\underset{\sigma \in S_{k}}{\sum }\lambda^{su,k}u_{\sigma }^{su,k}$$

$$x_{C}=\left\{p_{t}^{e,k},p_{t}^{e,sys},h_{t}^{e,k},h_{t}^{e,sys},h_{t}^{d},s_{t},s_{t}^{in},s_{t}^{out},d_{t},p_{\sigma ,\tau }^{e,k},h_{\sigma ,\tau }^{e,k},u_{\sigma }^{max,k},u_{\sigma }^{min,k},u_{\sigma }^{prot,k},u_{\sigma }^{on,k},u_{\sigma }^{off,k},u_{\sigma }^{su,k}\right\}$$

Constraints ([Disp-formula eq17]–[Disp-formula eq23]), which describe electrolyzer operation in scenario (B), are replaced by constraints ([Disp-formula eq27]–[Disp-formula eq32]) in scenario (C). Constraint ([Disp-formula eq27]) enforces that each device *k* can be either ON or OFF at every time slot σ, while constraints ([Disp-formula eq28]–[Disp-formula eq29]) define the corresponding start-up state at the σ-scale. When the device is in its ON state, it must exclusively select one operating mode as enforced by ([Disp-formula eq30]). Accordingly, its power consumption ([Disp-formula eq31]) and hydrogen production ([Disp-formula eq32]) are determined by combining the binary variables $\left\{u_{\sigma }^{prot,k},\;u_{\sigma }^{max,k},\;u_{\sigma }^{min,k}\right\}$ with device-specific parameters $P_{\tau }^{prot,k}$ , *P* ^*max*,*k*^, *P* ^*min*,*k*^, and the conversion coefficients *A* ^*k*^ and *B* ^*k*^.

17 $$1=u_{\sigma }^{on,k}+u_{\sigma }^{off,k},,\forall k\in K,\forall \sigma \in S_{k}$$

18a $$u_{\sigma }^{su,k}\geq u_{\sigma }^{on,k}-u_{\sigma -1}^{on,k},,\forall k\in K,\forall \sigma \in S_{k}\backslash \left\{1\right\}$$

18b $$u_{\sigma =1}^{su,k}=0,,\forall k\in K$$

19 $$u_{\sigma }^{on,k}=u_{\sigma }^{prot,k}+u_{\sigma }^{max,k}+u_{\sigma }^{min,k},,\forall k\in K,\forall \sigma \in S_{k}$$

20 $$p_{\sigma ,\tau }^{e,k}=u_{\sigma }^{prot,k}P_{\tau }^{prot,k}+u_{\sigma }^{max,k}P^{max,k}+u_{\sigma }^{min,k}P^{min,k},,\forall k\in K,\forall \sigma \in S_{k},\forall \tau \in N_{k}$$

21 $$h_{\sigma ,\tau }^{e,k}=A^{k}p_{\sigma ,\tau }^{e,k}+B^{k}u_{\sigma }^{on,k},,\forall k\in K,\forall \sigma \in S_{k},\forall \tau \in N_{k}$$

Similar to scenario (B), scenario (C) also adopts constraints ([Disp-formula eq9]–[Disp-formula eq15]) from scenario (A) to model hydrogen storage and demand. However, a key challenge arises because these constraints are formulated on the original *t*-scale, whereas electrolyzer operation in scenario (C) is scheduled on (σ, τ)-scale. To reconcile this mismatch, additional mapping constraints ([Disp-formula eq33]–[Disp-formula eq34]) link the two time domains. Using this mapping, the existing aggregation constraints ([Disp-formula eq24]–[Disp-formula eq25]) are then employed to compute the total power consumption and hydrogen production on the *t*-scale. This ensures that the operational decisions determined within the electrolyzer subproblem are consistently transferred to the hydrogen storage and demand components of the model.

22 $$p_{t}^{e,k}=p_{\sigma ,\tau }^{e,k},,where\;t=N_{k}\times \left(\sigma -1\right)+\tau ,\forall k\in K,\forall \sigma \in S_{k},\forall \tau \in N_{k}$$

23 $$h_{t}^{e,k}=h_{\sigma ,\tau }^{e,k},,where\;t=N_{k}\times \left(\sigma -1\right)+\tau ,\forall k\in K,\forall \sigma \in S_{k},\forall \tau \in N_{k}$$

Through this formulation, the key characteristics of the structured operation in scenario (C) can be clearly identified by comparison with the fully flexible operations in scenarios (A) and (B). This comparison is summarized in Table 1, which highlights the differences in how electrolyzer operation is determined across the three scenarios. In conventional scheduling formulations, the power consumption $p_{t}^{e,k}$ is modeled as a *continuous* decision variable bounded within allowable power limits. In contrast, under the proposed protocol-based framework, power consumption is determined entirely by a set of binary decision variables and therefore becomes a *discrete* choice. Similarly, whereas conventional approaches regulate operational flexibility primarily through ramping constraints, the proposed framework manages flexibility through the *definition of structured protocols* and the *dynamic selection of operating modes*.

**Table 1:** Summary of Constraints for Optimization Formulations of Scenarios (A–C)

		(A)	(B)	(C)
Electrolyzer	State transitions	([Disp-formula eq2]), ([Disp-formula eq3]), ([Disp-formula eq4])	([Disp-formula eq17]), ([Disp-formula eq18]), ([Disp-formula eq19])	([Disp-formula eq27]), ([Disp-formula eq28]), ([Disp-formula eq29])
	Operation constraints	Capacity: ([Disp-formula eq5]), ([Disp-formula eq6]) Ramping: ([Disp-formula eq7])	Capacity: ([Disp-formula eq20]), ([Disp-formula eq21]) Ramping: ([Disp-formula eq22])	Operating mode selection: ([Disp-formula eq30]), ([Disp-formula eq31])
	Hydrogen production	([Disp-formula eq8])	([Disp-formula eq23])	([Disp-formula eq32])
	Aggregation across units (parallel operation)		([Disp-formula eq24]), ([Disp-formula eq25])	([Disp-formula eq24]), ([Disp-formula eq25]), ([Disp-formula eq33]), ([Disp-formula eq34])
Hydrogen storage		([Disp-formula eq9]–[Disp-formula eq14])		
Hydrogen demand (daily)		([Disp-formula eq15])		

## 3 Case Studies

### 3.1 Setting

We conduct case studies using real power-grid data to evaluate the performance of different operating scenarios based on their respective mathematical formulations. Specifically, we examine how flexibility is implemented in each scenario, quantify flexibility using an economic metric such as electricity cost, and systemically compare flexibility across scenarios. Through scenarios (A) and (B) in Section 3.3, we investigate how the ramping ratio *R* governs system flexibility by manipulating its value under otherwise identical conditions. In addition, case studies for scenario (B) illustrate how parallel operation of multiple devices provides greater flexibility than single-device operation in scenario (A). Scenario (C), which is outlined by the proposed framework, demonstrates how operational flexibility can be adjusted through the combination of multiple structured protocols. To explore a broad range of operating strategies, we conduct case studies using several protocol sets and operating modes. The resulting analyses evaluate whether these dynamic combinations can still achieve a high degree of flexibility despite their inherently structured nature.

### 3.2 Data and Parameters

We consider a grid-connected electrolysis system with a total capacity of 10 MW. Real-time market (RTM) data from the Electric Reliability Council of Texas (ERCOT) for a single day (July 1, 2024) is used as the electricity price input, with a temporal resolution of 15 min. Accordingly, the scheduling time step *t* is set to 15 min to match the resolution of the given market data. Across all scenarios, we assume the same electrolyzer technology, an alkaline electrolyzer, to ensure a fair comparison. Alkaline electrolyzers have been widely deployed commercially due to their cost-effectiveness, reliability, and scalability.^4^ All fixed parameters used in each scenario are summarized in Table 2.

**Table 2:** Model Parameters Used in Scenarios (A–C)

	**Scenario**	**Parameter**	**Value**	**Unit**
Electrolyzer	(A)	*P* ^*max*^	10	MW
		*P* ^*min*^	1.5	MW
		λ^*su*^	150 (=*P* ^*max*^ × 15)	$
	(B), (C)	*P* ^*max*,*k*^	1	MW
		*P* ^*min*,*k*^	0.15	MW
		λ^*su*,*k*^	15 (=*P* ^*max*,*k*^ × 15)	$
	All	*A* = *A* ^*k*^	18.031	kg/MWh
		*B* = *B* ^*k*^	2.628	kg/h
Storage	All	*S* ^*ini*^	0	kg
		*C* ^*S*^	4327.44 (=*P* ^*max*^ × *A* × 24)	kg
		*C* ^*S*,*out*^	180.31 (=*P* ^*max*^ × *A*)	kg/h
Demand (Daily)	All	$$D_{i}^{min}$$	3245.58 (=*P* ^*max*^ × *A* × 24 × 0.75)	kg

Scenario (A) operates a single 10-MW electrolyzer, whereas scenarios (B) and (C) consist of ten 1-MW electrolyzers operating in parallel, such that the total installed capacity is evenly distributed across units. This design ensures that all scenarios are compared under the same hydrogen production capability. Most model parameters are adopted or scaled from previous studies,^21,22^ which developed a detailed scheduling model for an alkaline electrolyzer based on experimental data.^41,44^ The minimum operating load of each electrolyzer is set to 15% of its power capacity. Because all scenarios are assumed to use identical electrolyzer technology, the device-specific parameters *A* and *B* (or *A* ^*k*^ and *B* ^*k*^) associated with the linearized hydrogen production curve are uniformly applied across all scenarios. Although hydrogen production curves are inherently nonlinear, it is common practice to linearize them for analytical tractability in industrial-scale studies. Similarly, we assume an identical cold start-up cost parameter 15 $/MW for all individual electrolyzers,^45^ which is multiplied by the maximum power load *P* ^*max*^ (or *P* ^*max*,*k*^) of device to obtain its start-up cost λ^*su*^ (or λ^*su*,*k*^). In addition, all scenarios share the same storage- and demand-related parameters to ensure consistency across the comparison. Our framework is implemented in the Julia programming language and solved using Gurobi; all code and data needed to reproduce the results is available at https://github.com/zavalab/JuliaBox/tree/master/ElectrolysisProtocols.

### 3.3 Flexible Operation: Scenarios (A) and (B)

Using the basic scenario (A) for grid-connected electrolysis operation, we explore the role of the ramping ratio *R* as a controller of operational flexibility. The top panels of Figure 4 present the power consumption and hydrogen storage profiles in scenario (A) under different ramping conditions, R = 0.2, 0.5, and 1.0. As the ramping ratio increases, the constraints on power variations become less restrictive, allowing the electrolyzer to adjust its operating level more rapidly in response to electricity market variations. This behavior follows directly from the ramping constraint ([Disp-formula eq7]), which governs the operation of scenario (A) and becomes inactive when R = 1.0. Consequently, the case with the highest ramping ratio makes the greatest use of hydrogen storage by retaining surplus hydrogen produced during periods of low electricity prices. Similar trends are observed in the operation of scenario (B), which likewise becomes more flexible as the ramping ratio increases.

**Figure 4:**
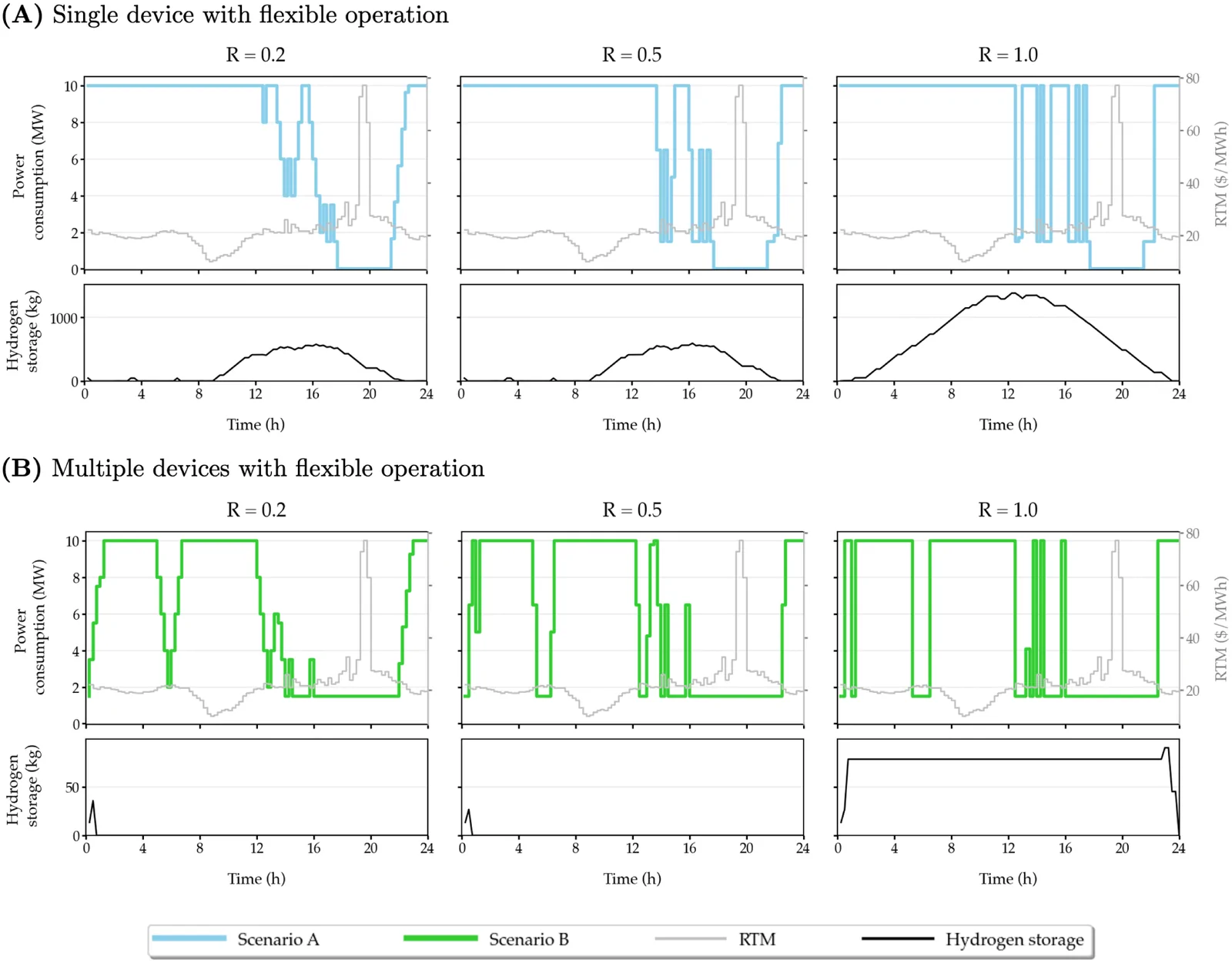
Total power consumption (colored lines) and hydrogen storage (black lines) profiles for flexible operating scenarios. Top panels show the profiles of a single 10-MW electrolyzer in scenario (A). Bottom panels show the aggregated profiles of ten 1-MW electrolyzers in scenario (B). Case studies for both scenarios use identical single-day real-time market (RTM, gray lines) data under different ramping conditions, with R = 0.2, 0.5, and 1.0.

The only structural difference between scenarios (A) and (B) lies in the number of electrolyzers operating in parallel; scenario (A) employs a single 10-MW unit, whereas scenario (B) consists of ten 1-MW modules with the same total capacity. The bottom panels of Figure 4 show that the parallel operation enables the system to remain at, or close to, its minimum load when the electricity price exceeds a threshold of approximately 20 $/MWh. This price threshold is enforced more tightly in the parallel operation than in the counterpart single-device cases. In scenario (A), the electrolyzer continues to operate near its maximum capacity during 0–12 h even when the price rises above 20 $/MWh. These results indicate that aggregating multiple smaller devices can provide greater operational flexibility than a single large device. Since the parallel configuration can respond more effectively to market dynamics, it may also reduce the required hydrogen storage capacity, which is a trend consistent with the storage profiles in Figure 4. Moreover, in scenario (B), all modules remain online throughout the operating horizon, thereby avoiding additional cold start-up costs.

From a mathematical perspective, the greater flexibility observed under parallel operation results from an *increased degree of freedom* in determining the aggregate power consumption $p_{t}^{e,sys}$ . This can be interpreted in two complementary ways. First, given that the minimum power load of each device is fixed at 15% of its capacity and all units can be operated independently, the parallel configuration effectively expands the feasible range of aggregate power consumption. In scenario (A), where a single 10-MW electrolyzer is used, the feasible range is constrained to $p_{t}^{e,sys}\in \left\{1.5,\;10.0\right\}$ . In contrast, in scenario (B), which consists of ten independent 1-MW units, the aggregate power can take any value within $p_{t}^{e,sys}\in \left\{0.15,\;10.0\right\}$ . This is possible because the system can operate a single unit at its minimum load of 0.15 MW while keeping the remaining units offline. This observation is consistent with a prior study^37^ that identifies the reduced effective lower bound of power consumption as a key mechanism through which parallel operation achieves higher flexibility. Second, in multidevice operation, ramping constraints are enforced at the unit level rather than at the aggregate level. In scenario (A), the single power variable $p_{t}^{e,sys}$ must evolve coherently over time under a system-wide ramping constraint. In scenario (B), however, power adjustments can be distributed across individual units: some units may ramp up, others may ramp down, while the remaining units may hold their states within the same time step. This behavior is illustrated by the individual electrolyzer trajectories for scenario (B) with R = 1.0 in Figure 5. Although the aggregate of the ten trajectories maps to a single variable $p_{t}^{e,sys}$ , as in scenario (A), the underlying feasible set is significantly expanded due to the independent movement of individual units. The resulting high degree of freedom relaxes constraints on power adjustments, leading to a more flexible operating profile.

**Figure 5:**
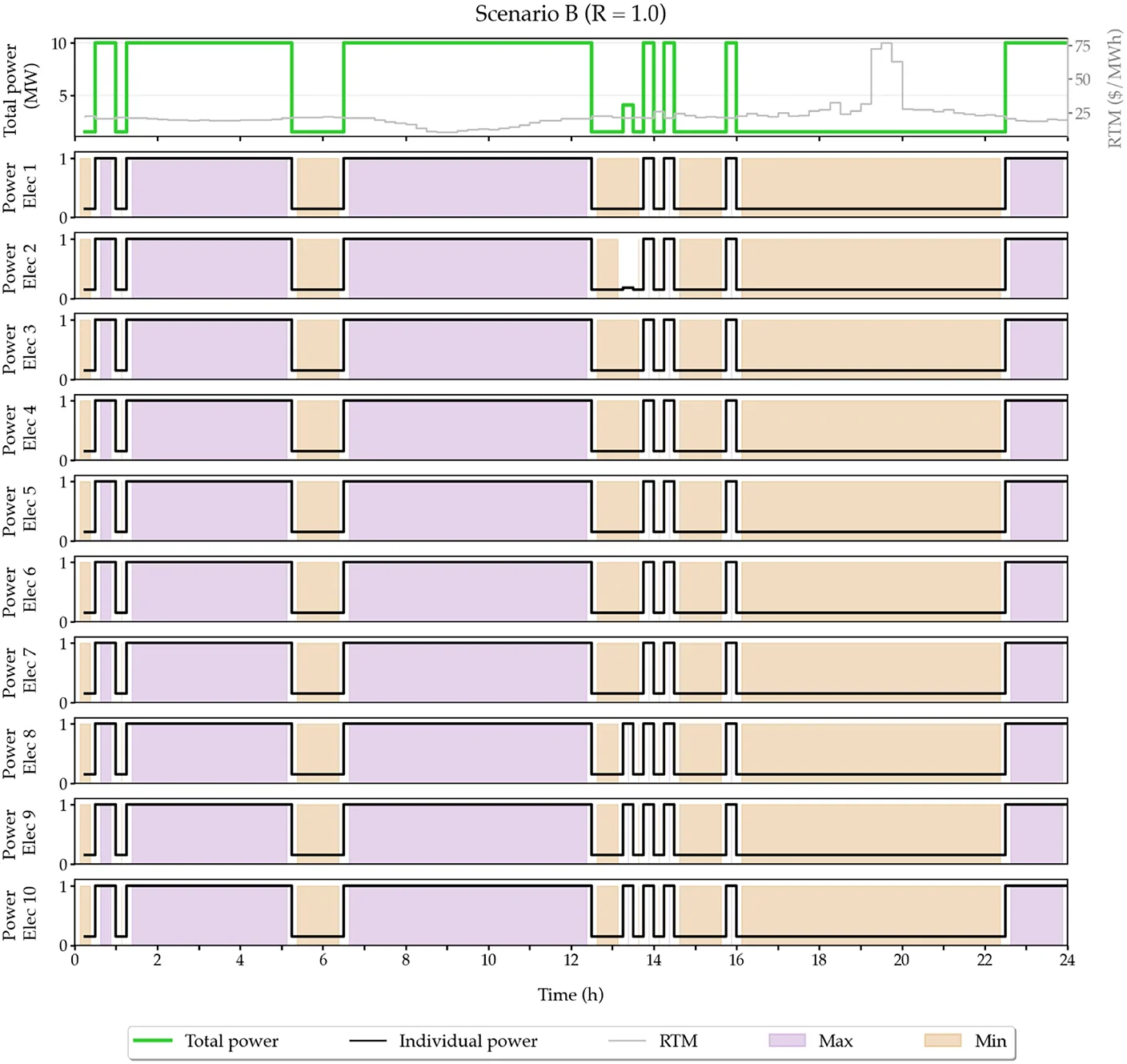
Disaggregation of total power consumption (top panel) into ten individual electrolyzer profiles for fully flexible operation in **scenario (B) with R = 1.0**. Colored regions in each individual profile indicate time periods during which each electrolyzer operates at its maximum or minimum power load.

Operational flexibility can also be represented using economic performance as a quantitative metric. In this study, the electricity cost obtained from the scheduling optimization problem for each case serves as the basis for comparing relative flexibility: lower electricity costs indicate greater operational flexibility. Figure 11 summarizes the resulting cost in terms of the normalized electricity cost, defined as the optimal electricity cost divided by the corresponding hydrogen production for each case. This metric can be interpreted as the contribution of electricity cost to the levelized cost of hydrogen (LCOH).

As references for comparison, we define two cases where a single 10-MW electrolysis system is forced to operate at constant power level. The first case, which is labeled with *P* = 10 MW, operates at its full capacity of 10 MW over the entire time horizon, thereby incurring the highest electricity cost. The second case labeled with *P* = 7.35 MW operates at the constant power level that achieves the same hydrogen production (demand) as scenarios (A) and (B). The constant value 7.35 MW is obtained by solving the optimization problem for scenario (A) with the ramping constraint ([Disp-formula eq7]) replaced by $p_{t}^{e,sys}=p^{const}$ . The new decision variable *p* ^*const*^ denotes the constant operation level over the entire horizon and its value is primarily decided by the demand constraint ([Disp-formula eq15]).

Adopting the constant-operation case with *P* = 7.35 MW as the baseline, as shown in the Figure 11, yields a fair comparison of scenarios based on the same hydrogen production. The percentage cost savings relative to the baseline are used to evaluate the economic performance and flexibility of each case. Consistent with previous studies,^13,46^ flexible operation reduces electricity cost relative to constant baseline operation by exploiting temporal variations in electricity prices. Comparing the implementation sets within scenarios (A) and (B), we observe that higher ramping limits generally lead to lower electricity costs, which is consistent with enhanced operational flexibility. Moreover, while single-device operation in scenario (A) shows at most 12.7% cost savings, even under fully flexible operation (R = 1.0), multidevice operation in scenario (B) attains 29.1%–29.4% cost savings regardless of the ramping ratio. This substantial difference indicates that parallel operation is an effective strategy for improving flexibility from an operational viewpoint.

### 3.4 Operation Using Structured Protocols: Scenario (C)

For scenario (C), square-wave structured protocols are predefined for each of the ten electrolyzers prior to optimization-based scheduling. Each protocol, represented by the set $P_{\tau }^{prot,k}$ , is characterized by its power load, period, and duty cycle over a prescribed inner-slot time domain $\tau \in N_{k}$ . In practical operation, operators have the flexibility to determine the slot length *N*_*k*_ for each unit *k* and to define the corresponding protocol within *N*_*k*_ according to operational preferences. For example, if more frequent adjustments in operating mode are required, the slot length can be reduced to achieve finer time resolution. Additionally, operators may adopt preferred protocols that offer practical advantages, such as improved durability or production efficiency.

As a representative operator, this work constructs three sets of square-wave protocols, as illustrated in Table 3 and Figure 6. The sets are distinguished by their slot lengths, which serve as key parameters in determining the scheduling time resolution. In set I, all ten protocols have a slot length of 2 h, whereas in set III, all protocols have a slot length of 1 h. As an intermediate case, set II consists of six protocols with 1-h slot lengths and four with 2-h slot lengths, demonstrating how protocols with different time resolutions can be combined. Given that each time step in the ERCOT RTM environment has 15 min interval, slot lengths of 2 and 1 h correspond to *N*_*k*_ = 8 and *N*_*k*_ = 4, respectively. The remaining protocol specifications for each set are summarized in Table 3 and Figure 6. The specification parameters are bounded such that $P^{min,k}\leq P_{\tau }^{prot,k}\leq P^{max,k}$ (Section 2.2.3), the period does not exceed the slot length, and the duty cycle lies between 0 and 1. Subject to these bounds, each protocol set is defined so that the ten electrolyzers are assigned distinct combinations of protocol parameters.

**Table 3:** Protocol Specifications for Scenario (C)

	**Protocols I**				**Protocols II**				**Protocols III**			
	Slot length (h)	Power load (MW)	Period (h)	Duty cycle	Slot length (h)	Power load (MW)	Period (h)	Duty cycle	Slot length (h)	Power load (MW)	Period (h)	Duty cycle
Elec 1	2 (*N*_*k*_ = 8)	1	0.5	0.5	1 (*N*_*k*_ = 4)	1	0.5	0.5	1 (*N*_*k*_ = 4)	1	0.5	0.5
Elec 2		0.5	0.5	0.5		0.5	0.5	0.5		0.5	0.5	0.5
Elec 3		1	1	0.5		1	1	0.5		1	1	0.5
Elec 4		0.5	1	0.5		0.5	1	0.5		0.5	1	0.5
Elec 5		1	1	0.75		1	1	0.75		1	1	0.75
Elec 6		1	1	0.25		1	1	0.25		0.5	1	0.75
Elec 7		1	2	0.5	2 (*N*_*k*_ = 8)	1	2	0.5		1	1	0.25
Elec 8		0.5	2	0.5		0.5	2	0.5		0.5	1	0.25
Elec 9		0.5	2	0.75		0.5	2	0.75		1	1	0.5
Elec 10		0.5	2	0.25		0.5	2	0.25		0.5	1	0.5

**Figure 6:**
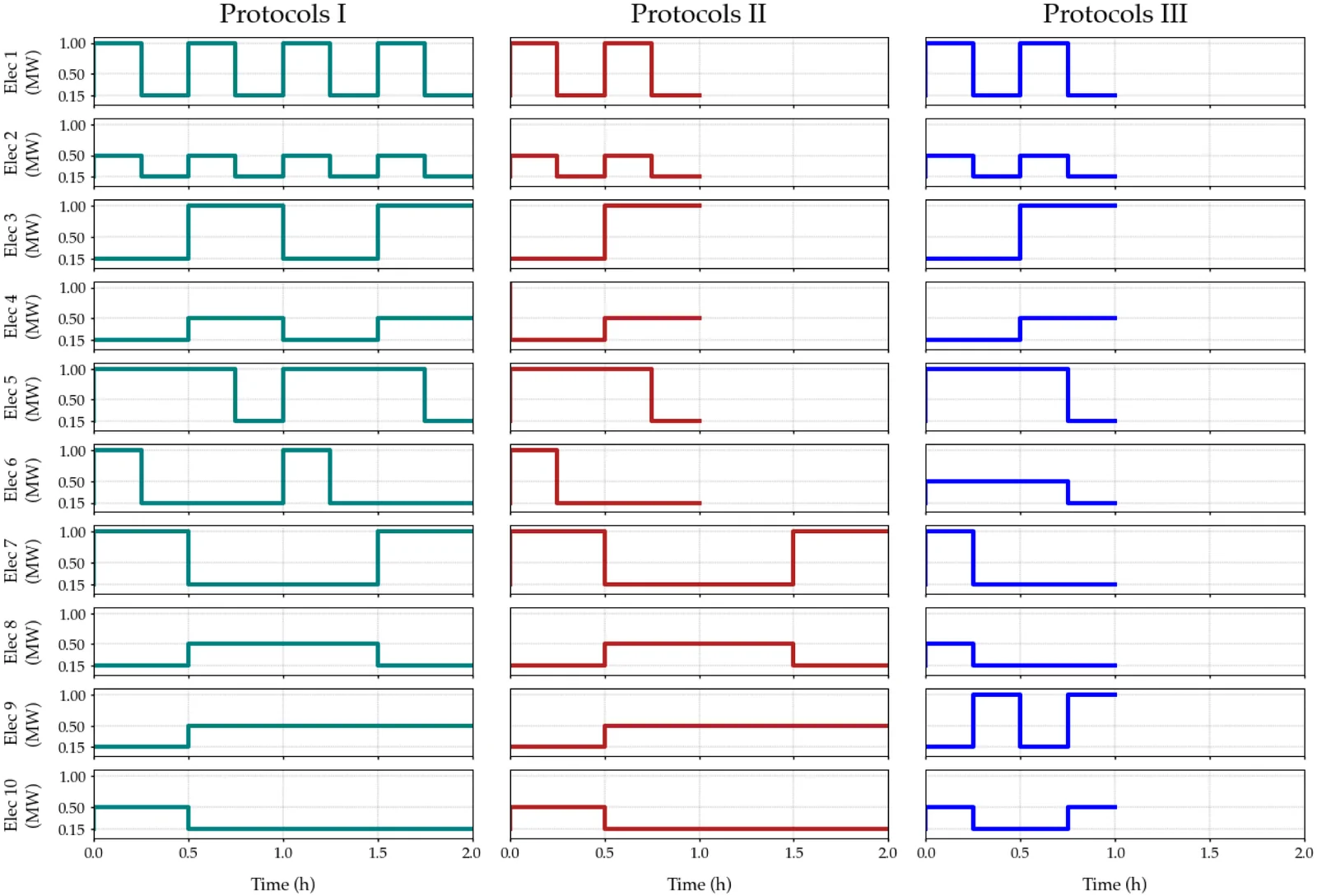
Plots of three sets of square-wave protocols for ten electrolyzers in scenario (C), as specified in Table 3. The sets primarily differ in the slot lengths assigned to each electrolyzer.

In addition to considering diverse protocol sets, we examine two different case studies for each protocol set in order to isolate and highlight the impact of protocol trajectories. In the first case, all devices are restricted to operating only at their maximum or minimum power levels, without selecting their assigned structured protocols (Figure 7a). In the second case, conversely, devices are allowed to choose among three operating modes: operating at maximum power, operating at minimum power, or following their assigned structured protocols (Figure 7b). Although the mathematical formulation presented in Section 2.2.3 corresponds to the latter case, the former case can be obtained with a simple modification by removing the terms associated with $u_{\sigma }^{prot,k}$ from constraints ([Disp-formula eq30]) and ([Disp-formula eq31]). These constraints are then replaced by ([Disp-formula eq35]) and ([Disp-formula eq36]), while all other constraints remain unchanged. Under this setting, the predefined time-slot length *N*_*k*_ for each electrolyzer becomes the sole factor influencing dynamic behavior, as the specific protocol is no longer considered.

24 $$u_{\sigma }^{on,k}=u_{\sigma }^{max,k}+u_{\sigma }^{min,k}$$

25 $$p_{\sigma ,\tau }^{e,k}=u_{\sigma }^{max,k}P^{max,k}+u_{\sigma }^{min,k}P^{min,k}$$

**Figure 7:**
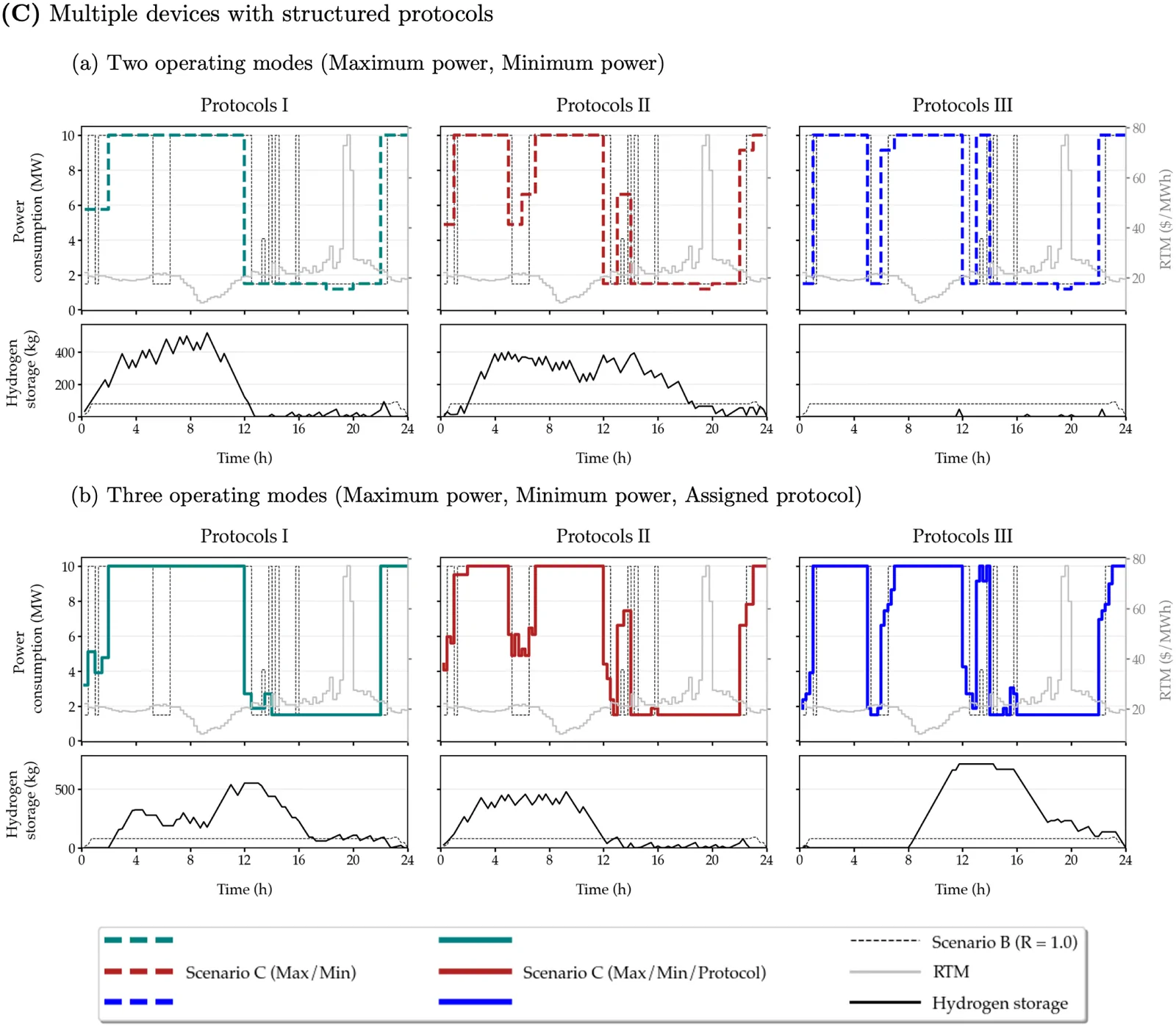
Total power consumption (colored lines) and hydrogen storage (black lines) profiles for protocol-based operating scenarios (C) using three protocol sets I, II, and III. Top panels (a) show operation restricted to two modes, operating only at maximum or minimum power load. Bottom panels (b) show operation with three modes, including an additional structured-protocol mode. All six case studies use identical single-day real-time market (RTM, gray lines) data, which are the same as those used in Figure 4. Across all cases, the fully flexible operation in scenario (B) with R = 1.0 serves as the reference trajectory (black dotted lines) for both power consumption and hydrogen storage profiles.

The six case studies, including the two operating schemes for each of the three protocol sets, are designed to conceptually reflect the expected degradation characteristics associated with different operating profiles. Since the degradation rate is reported to be lowest at the minimum and maximum power loads,^35,36^ restricting the operating modes to these two extreme power levels, regardless of whether structured protocols are employed, is expected to take advantage of the favorable degradation behavior. Particularly, the degradation rate typically peaks at intermediate-to-low power levels across a wide range of operating conditions, including temperature^35^ and pressure.^36^ Thus, selecting a protocol power level of 0.5 MW in this framework is expected to avoid this degradation peak. In addition, the proposed structured protocols are designed online, avoiding OFF and start-up states within each time slot. This not only eliminates repeated start-up costs but is also expected to reduce degradation, as the frequent startup/shutdown events^47^ and the resulting OFF (idle) states^48^ are generally associated with accelerated electrolyzer degradation.

Figure 7 presents the results of all case studies in terms of their time-varying power consumption and hydrogen storage profiles. The reported power consumption represents the aggregate load of all ten electrolyzers in the system. As a reference, we define the case of scenario (B) with R = 1.0 as *fully flexible operation*, which shares the same system configuration as scenario (C). The reference operation is also plotted in Figure 7 as a black dotted line behind the operational trajectory of each case. This corresponds to the optimal operation achievable through parallel operation of ten 1-MW electrolyzers and thus yields the lowest electricity cost, as shown in Figure 11. Therefore, the goal of scenario (C) is to approximate the reference trajectory by combining the structured protocols assigned to individual devices.

#### 3.4.1 Approximation of Fully Flexible Operation

The top panels of Figure 7 demonstrate the approximation through electrolysis operation profiles, where the profiles of scenario (C) show similar trajectories as the fully flexible profile across all six cases. Thus, a key observation holds across all of them: the aggregate of operating profiles approximate fully flexible operation. This observation is further quantified through electricity cost comparisons, as shown in Figure 11. Relative to the single-device operation in scenario (A), multidevice operation in scenarios (B) and (C) attains substantially greater cost savings due to enhanced flexibility, irrespective of whether a fully flexible or structured operating strategy is employed. Although the six cases within scenario (C) exhibit minor cost differences among themselves, they all deliver significant savings relative to the baseline case, approximately ranging from 28.6% to 29.0%. These values are comparable to the 29.4% cost savings achieved by the fully flexible operation (R = 1.0) in scenario (B). The principal finding obtained from this analysis is that even cases that restrict operation to only maximum and minimum power levels still provide meaningful cost savings, which is not overly different from the cases including protocols. This result suggests that even highly simplified operating strategies can retain substantial flexibility benefits over the baseline operation, highlighting the significance of parallel aggregation in achieving high flexibility.

Beyond the similarities among the cases, distinct effects of the different protocol sets can also be identified. Comparing the top panels of cases (a) and (b) of Figure 7, we demonstrate that allowing electrolyzers to select structured protocols in addition to operating at maximum or minimum power levels results in power consumption trajectories that more tightly approximate the fully flexible profile. Such enhanced flexibility arises from the combination of protocol diversity and parallel aggregation, whereby aggregating ten distinct protocols produces a denser and more varied set of small power adjustments at the system level. Furthermore, comparing the results obtained using protocols sets I, II, and III, each featuring progressively shorter average slot lengths, we observe that the aggregate operating trajectories increasingly tend to approach the fully flexible reference. This observation is consistent with the general expectation that scheduling at a finer temporal resolution enables more responsive and flexible control. The number of parallel electrolyzer units is also expected to influence the achievable flexibility, as a larger number of units can provide finer aggregate operating capacities and therefore a closer approximation to fully flexible operation. Exploring the relationship between the number of parallel units and the achievable flexibility remains an important direction for future research.

The optimal power consumption profiles lead to the corresponding hydrogen storage trajectories, as shown in the bottom panels of Figure 7. The storage profile can be interpreted as a direct consequence of optimal electrolysis operation. For example, the storage trajectory under Protocols I reflects its relatively coarse operational adjustments resulting from longer protocol lengths, compared with those under Protocols II and III. This behavior is readily observed in cases (a): Protocols I case exhibits a relatively simple charge–discharge cycle that closely follows its power consumption profile, Protocols II case produces a more distributed charging and discharging pattern throughout the day, and Protocols III case enables the most responsive storage trajectory. Furthermore, comparison of the maximum storage utilization in Table 4 shows that cases (b) consistently achieve greater hydrogen storage utilization than their corresponding cases (a), reflecting the additional operational flexibility provided by the structured protocols. Despite these overall trends, however, the detailed storage trajectories differ considerably among the six cases. Given that the optimal electricity costs of all cases in scenario (C) are comparable, this demonstrates that similar economic performance can be attained through multiple near-optimal operation schedules under identical demand constraints.

**Table 4:** Results Summary of Scenario (C) Compared to Fully Flexible Reference^a^

	**Protocols I**		**Protocols II**		**Protocols III**		
	(a)	(b)	(a)	(b)	(a)	(b)	scenario (B) (R = 1.0, reference)
Electricity cost ($)	11,229	11,178	11,178	11,159	11,176	11,157	11,099
Hydrogen production (kg)	3,250.9	3,245.7	3,246.3	3,245.7	3,246.3	3,245.7	$$3245.58\left(=D_{i}^{min}\right)$$
Maximum storage utilization (kg)	519	550	402	477	45	713	90
Number of start-up states	2	0	2	0	2	0	0

a (a) denotes the case of two-operation modes (Max/Min) and (b) denotes the case of three-operation modes including assigned protocols (Max/Min/Protocol).

#### 3.4.2 Electricity Price Arbitrage

The mechanisms underlying the aggregation of multiple electrolyzers are elucidated by analyzing the individual operating profiles of the ten units participating in the system, as visualized in Figures 5, 8, 9 and 10. Figure 5 presents the reference case for scenario (B) with R = 1.0, in which the power trajectory can be determined by directly tracking the electricity market prices without ramping restrictions. In contrast, Figures 8–10 illustrate the structured operation in scenario (C) using protocol sets I–III. Across all figures, the top panel (a) corresponds to operation with only maximum- and minimum-power modes, while the bottom panel (b) includes an additional structured-protocol mode. The dotted vertical grid lines in individual profiles indicate the time slots assigned to each device, allowing for a clear visual distinction between protocol-based structured operation (Figures 8–10) and conventional flexible operation (Figure 5).

**Figure 8:**
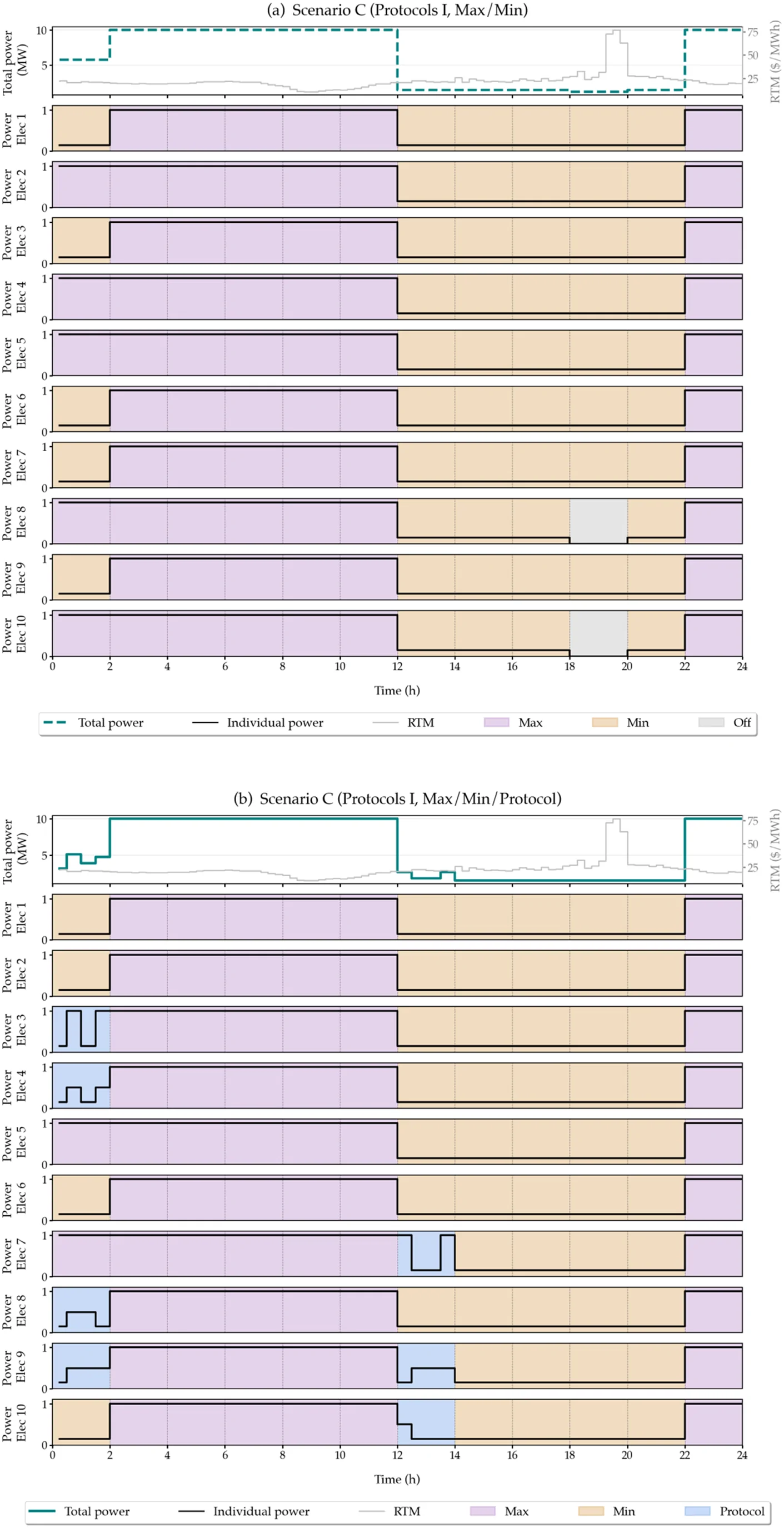
Disaggregation of total power consumption into ten individual electrolyzer profiles for **scenario (C) using protocol set I**: (a) two-mode operation; (b) three-mode operation including structured protocols.

**Figure 9:**
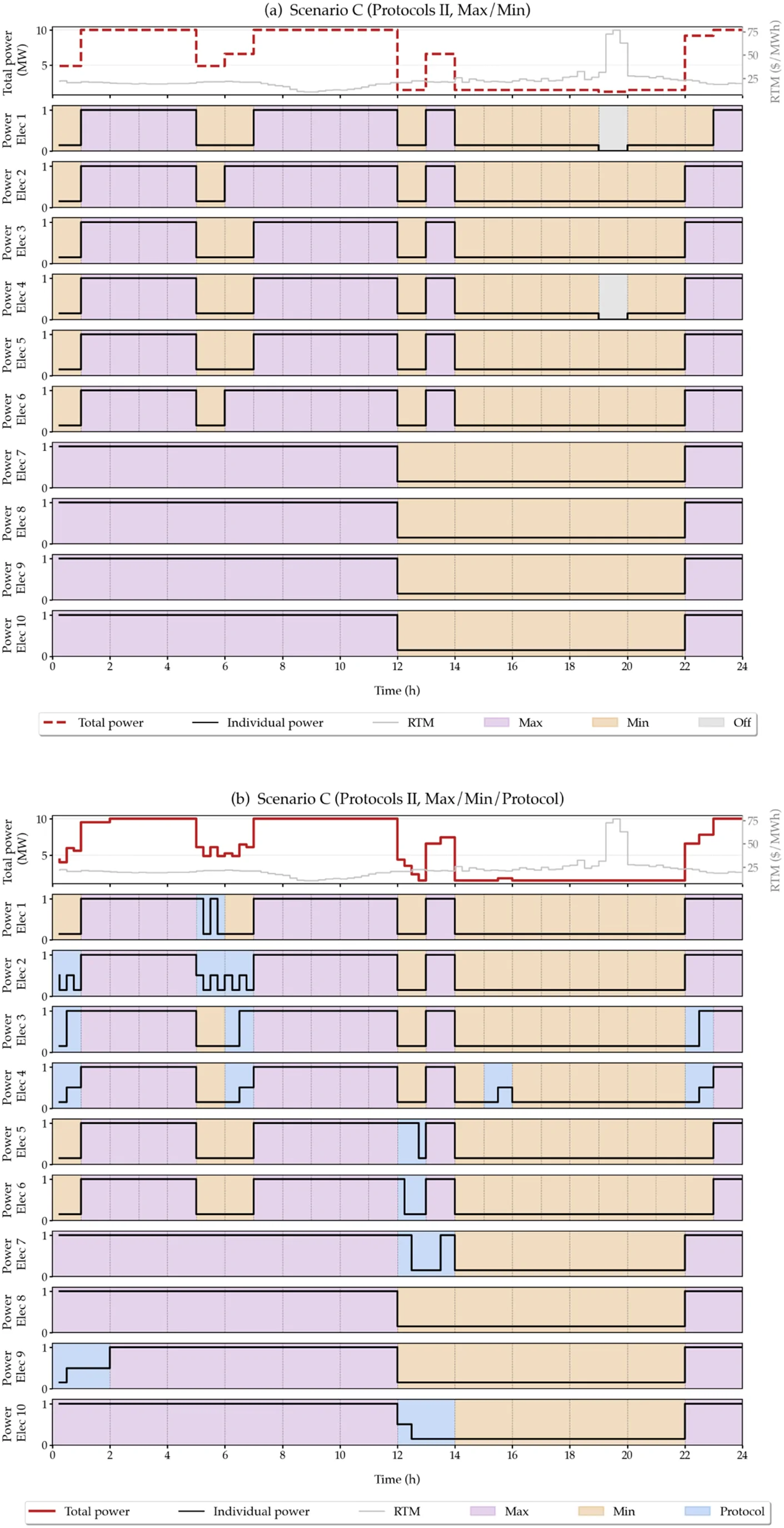
Disaggregation of total power consumption into ten individual electrolyzer profiles for **scenario (C) using protocol set II**: (a) two-mode operation; (b) three-mode operation including structured protocols.

**Figure 10:**
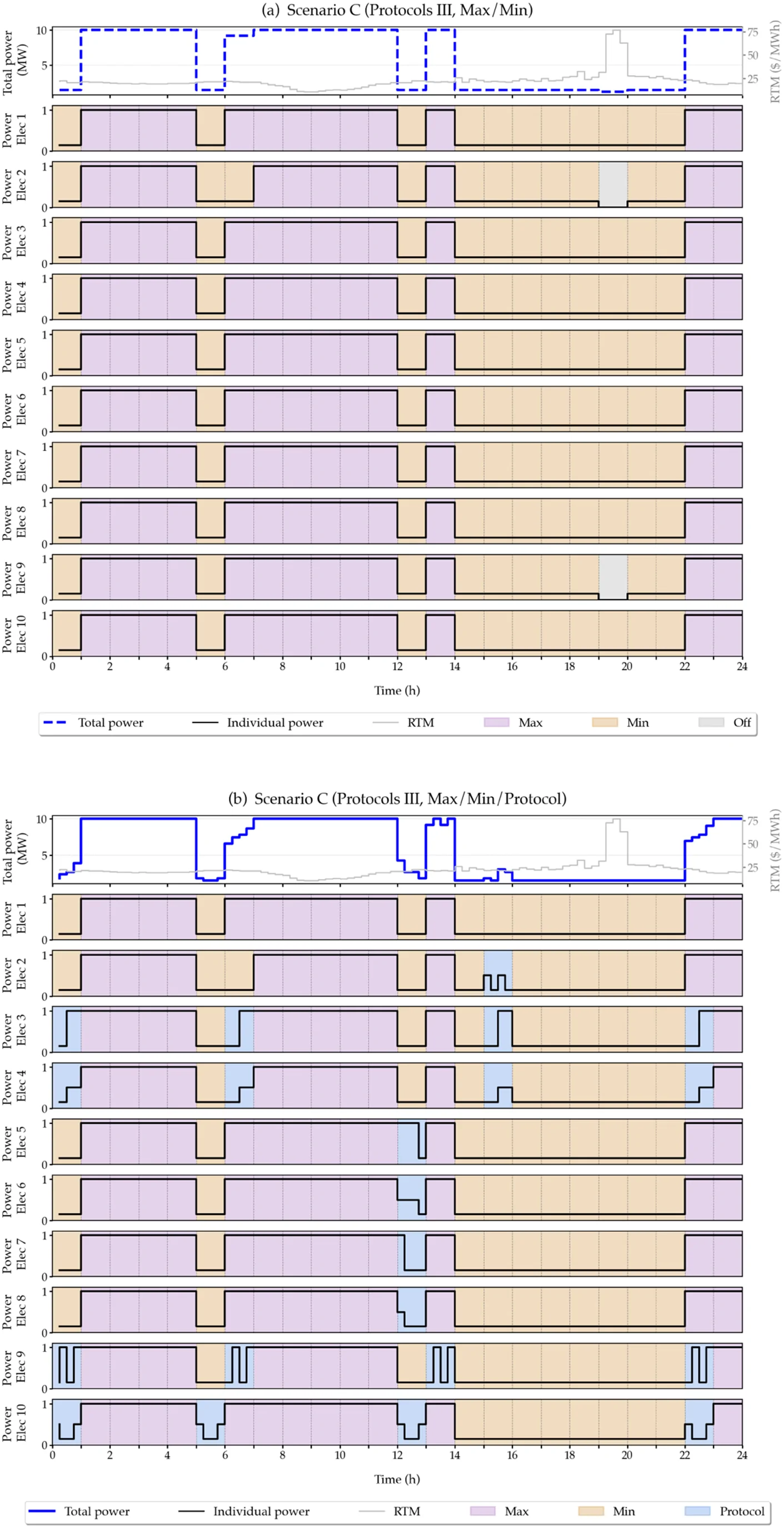
Disaggregation of total power consumption into ten individual electrolyzer profiles for **scenario (C) using protocol set III**: (a) two-mode operation; (b) three-mode operation including structured protocols.

Figure 5 shows that individual electrolyzers frequently operate at either their maximum or minimum power levels. This behavior reflects a typical response of grid-connected devices, which operate at maximum capacity during low-price periods and maintain minimum load during price peaks. This pattern explains why operating with only two modes, maximum- and minimum-power modes, can still be effective in leveraging electricity market conditions, despite the highly restrictive nature of this strategy. This tendency is clearly depicted in the top panel (a) of Figures 8–10.

When structured-protocol modes are introduced, the bottom panel (b) exhibits more refined and elaborate adjustments in operational trajectories through diverse combinations of operating modes. Across all protocol sets for cases (b) in scenario (C), common time intervals emerge, including 2–4 h, 8–12 h, and 16–22 h, during which all devices operate at either maximum or minimum power load. These periods indicate that the system continues to exploit low electricity prices or avoid high prices in a manner consistent with fully flexible operation. In contrast, during periods where fully flexible operation displays rapid and pronounced power changes in Figure 5, such as 0–2 h and 12–16 h, these fluctuations are instead approximated through combinations of predefined yet diverse protocols within or near the same intervals in Figures 8–10. Consequently, the proposed framework in scenario (C) captures market variability by replacing ramping-based flexibility control with dynamic combinations of structured protocols. Another advantage of introducing structured protocols into cases (a) is that it provides sufficient flexibility for all devices to remain online throughout the operating horizon in this study, thereby eliminating additional start-up costs and potentially mitigating degradation. This behavior is consistently observed across all protocol sets, as summarized in Table 4.

This substitution of continuous ramping into combinations of structured protocols is consistent with the mathematical formulations summarized in Table 1. In scenario (B), power adjustment is governed by ramping constraints on continuous variables, whereas in scenario (C), it emerges from the aggregation of discrete operating modes. Hence, the operational dynamics are determined by the protocol designs, as demonstrated by profile differences under protocol sets I, II, and III in the bottom panels (b) of Figures 8–10. These observations motivate the investigation of a broader class of structured protocols in future work, including triangular and Gaussian-shaped waveforms, as suggested in Section 2.2.3. Such protocols are expected to provide an even closer approximation to fully flexible operation because they inherently incorporate ramping behavior that square-wave protocols lack.

#### 3.4.3 Capital Cost Considerations

Despite advantages of protocol-based framework in achieving flexible operation and exploiting market arbitrage, the results also suggest potential increases in capital investment, as indicated by Table 4 and the hydrogen storage profiles in Figure 7. Although the optimization framework assumes a fixed hydrogen storage capacity (*C* ^*S*^ = 4327.44 kg), the storage level remains well below this limit in all cases. Therefore, the maximum storage utilization obtained from the optimization can be interpreted as an estimate of the minimum storage capacity required for each operating strategy. Compared with the fully flexible reference in scenario (B) with R = 1.0, the protocol-based cases in scenario (C) generally exhibit hydrogen overproduction and increased utilization of hydrogen storage. These findings suggest that protocol-based operation may require larger electrolysis and hydrogen storage capacities to achieve a comparable level of operational flexibility, thereby increasing the associated capital investment.

While this work evaluates the economic performance from an operational perspective, particularly in terms of electricity cost, the potential increase in capital cost motivates a comprehensive techno-economic assessment (TEA) that jointly considers both capital and operating expenditures over the system lifetime. Furthermore, the reduced degradation expected from structured protocols, along with the resulting extension of electrolyzer stack lifetime, should be incorporated into the TEA to provide a more holistic evaluation of the proposed framework.

#### 3.4.4 Robustness across Different Days

Since the initial case studies are conducted for a single day, we further examined the proposed approach across multiple days to demonstrate its broader applicability. Figure 12 summarizes the results for seven consecutive days (July 1–7, 2024) under the identical daily hydrogen demand. The top panel shows the actual ERCOT RTM prices over this period, while the bottom panel presents the corresponding normalized electricity costs for several operating strategies: the baseline case with *P* = 7.35 MW, a fully flexible operation in scenario (A) with R = 1.0, a fully flexible parallel operation in scenario (B) with R = 1.0, and structured-operation cases in scenario (C) using protocol sets I, II, and III. For all scenario (C) cases, electrolyzers are allowed to select among three operating modes: operation at maximum load, at minimum load, or following assigned structured protocols. Except for the electricity market data, all other system configurations and parameters remain identical to those used in the single-day scheduling study (Figure 11).

**Figure 11:**
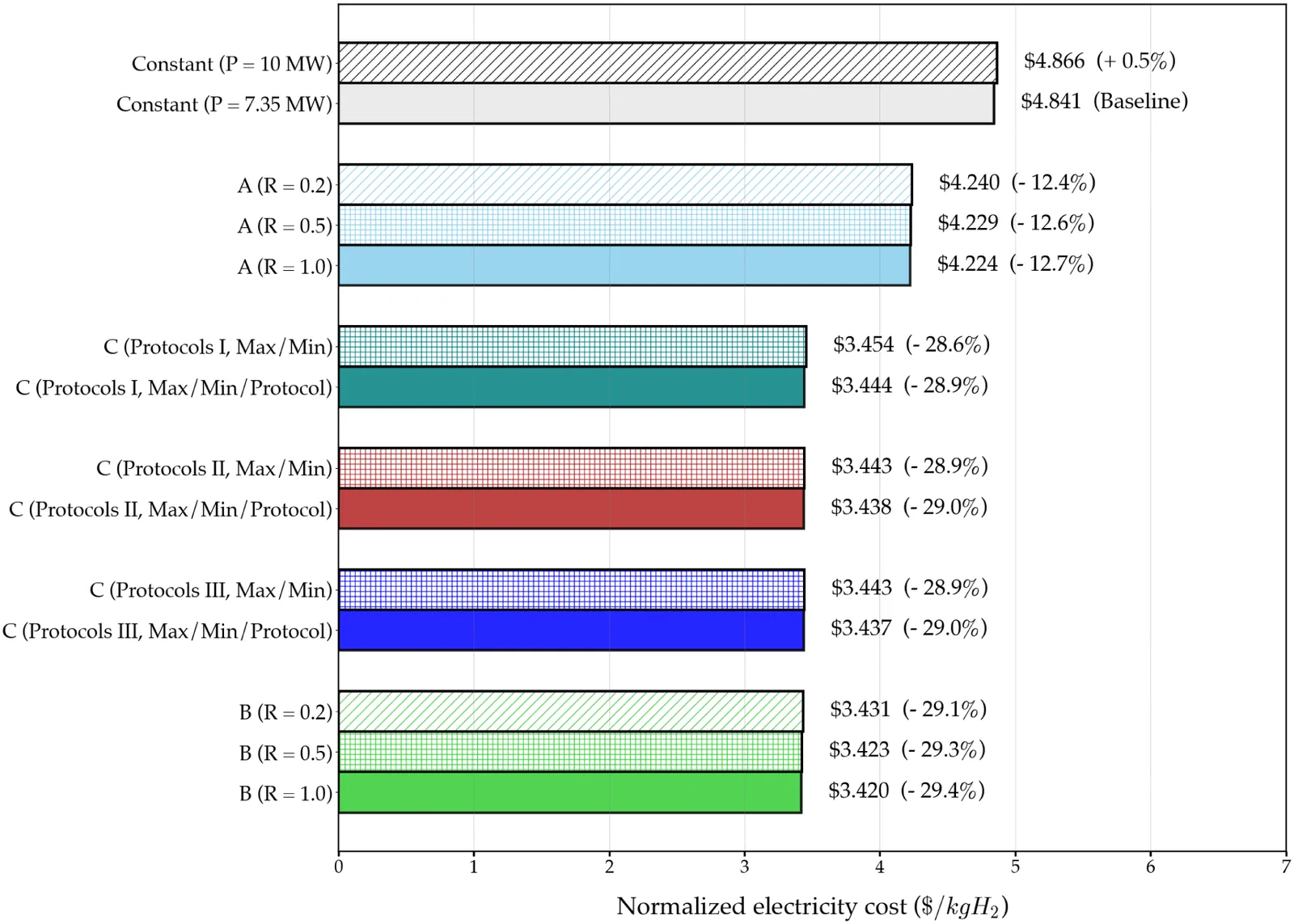
Summary of normalized optimal electricity costs for all cases addressed in Figures 4 and 7, which use identical single-day real-time market (RTM) data. This metric is obtained by dividing the optimal electricity cost by the corresponding hydrogen production for each case. Negative percentage values indicate cost savings relative to the baseline case, where a single 10-MW electrolyzer is forced to operate at a constant power level of *P* = 7.35 MW. This value was chosen to satisfy the same hydrogen demand as all other scenarios.

By jointly examining the top and bottom panels of Figure 12, we observe that the baseline case is entirely driven by the given market prices, as it operates continuously at constant capacity without any market-responsive adjustments. Although the baseline costs present substantial variations according to market data, the relative cost savings achieved by the other operating strategies exhibit consistent trends across different days, which are complemented with two observations. First, parallel operation of multiple electrolyzers consistently incurs lower electricity costs than single-device operation, reflecting its higher degree of operational flexibility. This is evident from the persistent cost gap between scenario (A) and scenarios (B) and (C) across all days. Second, within the parallel operation framework, structured operation in scenario (C) achieves cost savings and high flexibility comparable to those of fully flexible operation in scenario (B), as highlighted by saving plots. Overall, the consistency of results across this multiday analysis supports the conclusion that the proposed operating paradigm, based on the aggregation of structured protocols, can attain a high degree of operational flexibility under a wide range of electricity market conditions.

**Figure 12:**
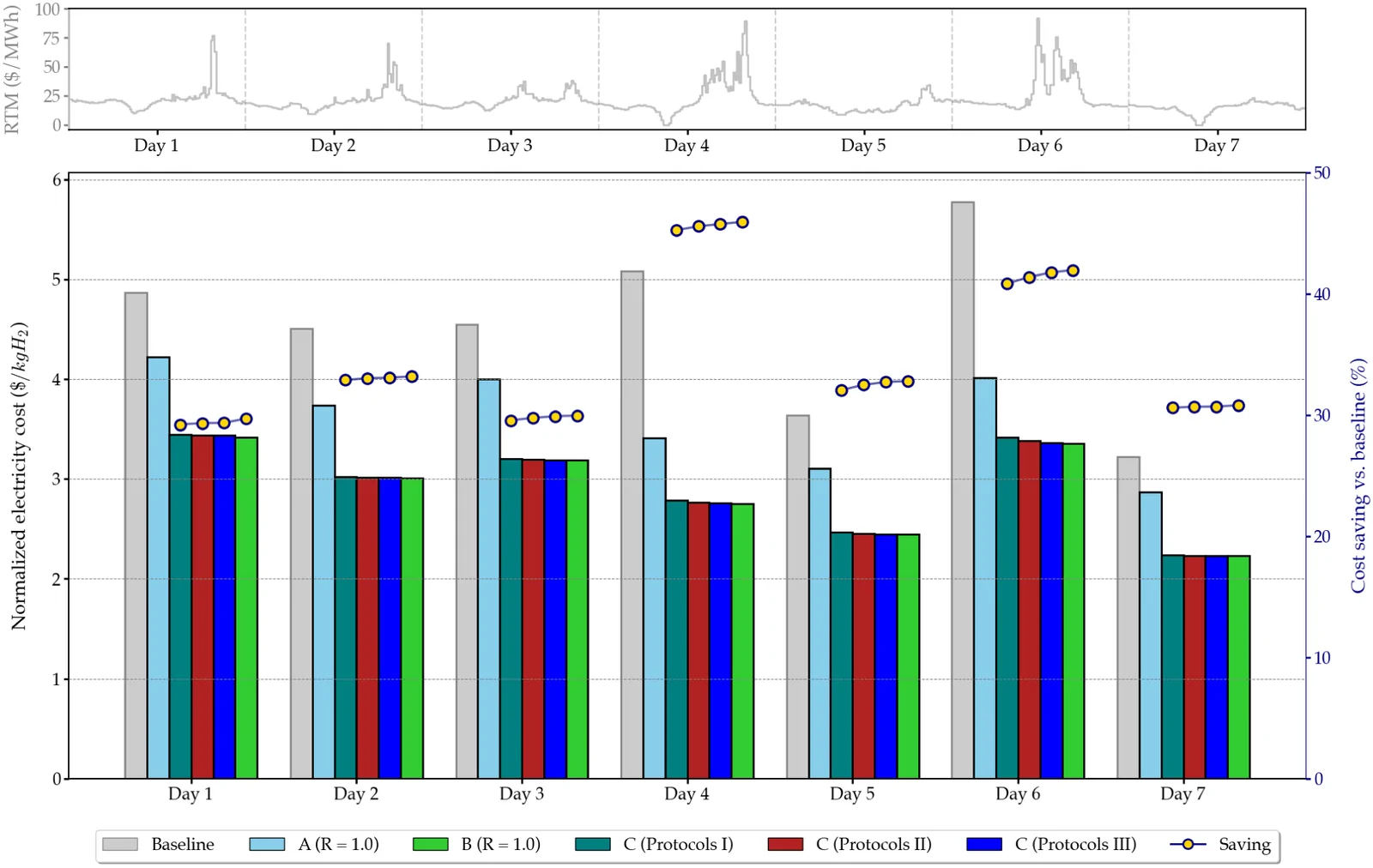
Summary of optimal electricity costs over 7 days. The top panel shows the real-time market (RTM) data, while the bottom panel presents the normalized electricity costs for multiple operating cases on each day. Baseline represents the operation at a constant power load *P* = 7.35 MW, which satisfies the same hydrogen demand constraint as the other scenarios. Scenarios (A) and (B) are implemented under fully flexible operation (R = 1.0). Scenario (C) is implemented using protocol sets I, II, and III, with all cases allowing three operating modes. The dots in the bottom panel, referenced to the right *y*-axis, represent the percentage cost savings achieved through parallel operation in scenarios (B) and (C) relative to the baseline case.

## 4 Conclusions and Future Work

The objective of this study was to propose a new operating paradigm that can enhance the load flexibility of grid-connected electrochemical systems while simultaneously mitigating degradation, thereby addressing the inherent trade-off between flexibility and durability. To this end, we introduce the dynamic combination of structured protocols as a new operational strategy. Structured protocols are expected to reduce degradation despite their limited flexibility at the individual device level. We hypothesize that the resulting loss of flexibility can be recovered through the parallel aggregation of the structured protocols, as their combined operation can approximate the behavior of a conventionally fully flexible system. To test this hypothesis, we construct and implement a series of case studies for electrolysis systems using real power grid data.

A comparison between a single large system and multiple smaller systems operating under the same total capacity and ramping-based control highlights the advantage of parallel operation in achieving high operational flexibility. The degree of flexibility can be quantified as electricity cost savings per amount of hydrogen produced, relative to the baseline operation where the system continuously operates at constant capacity under the same hydrogen demand. Through fully flexible operation, the multidevice system attains 29.4% cost savings over the baseline, while the single-device system reports 12.7% savings. The enhanced flexibility of multidevice system results from the increased degree of freedom in operation, which effectively relaxes operational constraints.

The flexibility benefits of parallel operation also enable a high degree of operational flexibility even when individual units operate under structured protocols. Even min-max cases with oversimplified protocols show higher approximation performances with 28.6%–28.9% cost savings, closely approximating the 29.4% savings attained under fully flexible operation. This highlights the significance of parallel aggregation of structured operation in achieving high flexibility. To better exploit its potential, different structured protocols are assigned to individual devices to replace the conventional ramping-based flexibility adjustments with the combination of distinct structured protocols. This allows the system to better leverage electricity market variations, by slightly increasing the cost savings to 28.9%–29.0% compared to the min-max counterpart cases. Overall results indicate that protocol-based operation can still achieve a high degree of flexibility comparable to ideally flexible operation. The proposed approach exhibits consistent trends across different days, demonstrating its robustness. These findings provide an important insight into the control and operation of multidevice systems: achieving strictly optimal flexibility is not always necessary, as it can be closely approximated through the combination of less flexible operating strategies.

While this work assesses the economic performance of protocol-based operation from an operational perspective, the potential increase in capital investment, together with the expected reduction in degradation and longer electrolyzer stack lifetime, motivates a comprehensive TEA. Furthermore, explicitly modeling auxiliary components, particularly compressors, would enable a more complete evaluation of plant-wide operational flexibility and techno-economic performance, as these components may not only contribute to capital expenditure but also introduce additional operational constraints and electricity consumption.^30,46^

A further direction for future work concerns the electrolyzer efficiency model. The present study assumes a constant electrolyzer efficiency through the linearized hydrogen production models ([Disp-formula eq8]), ([Disp-formula eq23]), and ([Disp-formula eq32]). Although this assumption is widely adopted in industrial-scale scheduling studies, its application to the parallel aggregation of small electrolyzer units may not fully capture the nonlinear efficiency characteristics of alkaline electrolyzers at low and high power levels,^41^ potentially leading to optimistic economic estimates. Moreover, because each structured protocol inherently prescribes a distinct operating profile across power levels, load-dependent efficiency may produce distinct efficiency profiles among protocols and thereby affect their economic performance. Thus, the preliminary analysis presented in the Supporting Information (SI) gauges the potential economic impact of nonlinear hydrogen production and, more importantly, highlights the need to incorporate a more accurate production model directly into the scheduling optimization. Such models, including piecewise-linear approximations^21^ or conic formulations,^22^ would enable a more realistic assessment of the economic benefits of protocol-based operation.

Building on the proposed framework, another important direction for future work is to systematically investigate how structured protocols should be designed for specific electrochemical systems. As discussed earlier, diverse patterns beyond the square-wave profiles considered in this work can serve as the basis for structured protocols, and different devices may favor different protocol designs depending on their material properties, operational characteristics, and economic and environmental objectives. Protocol design may account for the load-dependent efficiency and degradation behavior of the system, as briefly discussed in the SI, as well as additional operational requirements associated with safety. For example, H_2_ crossover in electrolyzers can become significant at low power loading and thus may impose a minimum operating load level to maintain product gas purity within safety limits.^49,50^ Because gas impurities can be influenced by electrolyte circulation, structured protocol design may account not only for the prescribed load levels but also for their coordination with additional operational strategies, such as appropriately timed switching between electrolyte circulation modes, to mitigate crossover.^51^ Thus, future work may extend structured protocol design beyond the selection of underlying patterns and load levels based on device-specific performance to incorporate supplementary operating strategies that address safety requirements.

Given that structured protocols were introduced in this work based on their potential for high durability, demonstrating their ability to achieve high flexibility further highlights the importance of verifying their durability. While device durability and operational flexibility have traditionally been treated separately, this work positions structured protocols as a unifying bridge between the two aspects. Structured protocols serve as the operational basis for degradation experiments to investigate how different protocol designs affect durability. At the same time, they are incorporated into the proposed operational framework to determine device schedules with a high degree of operational flexibility. A key contribution of this framework is that it not only approximates ideally flexible operation but also quantifies the associated flexibility loss as an economic metric relative to the optimal benchmark. This provides a foundation for integrating flexibility and durability within a unified economic analysis. In particular, once the degradation characteristics associated with specific protocols become available, it will be possible to assess whether the flexibility loss is offset by durability gains. In this way, the proposed framework establishes a pathway toward a holistic evaluation of electrochemical system operation, providing new insights into balancing flexibility and durability and ultimately helping to overcome their inherent trade-off.

## Supplementary Material



## Notes

Economic impact of nonlinear hydrogen production (PDF)

## Nomenclature

Variables are denoted by lower-case letters with binary variables specifically being denoted by letters *z* and *u*.: Parameters are denoted by upper-case letters, except for electricity prices and startup costs denoted by Greek letters λ and electricity market time resolution denoted by μ. Parameters and variables specified for each scenario are only used within that scenario. Otherwise, they are used across all scenarios.

## Indices and sets

t∈T: Set of time steps *t*
k∈K: Set of electrolyzers *k*
i∈I: Set of demand subperiods *i*
$T_{i}^{d}\subseteq T$: Set of time steps in demand subperiod *i*
$\sigma \in S_{k}$: Set of time slots σ for electrolyzer *k*
$\tau \in N_{k}$: Set of inner-slot time steps τ for electrolyzer *k*

## Parameters

$\lambda_{t}^{e}$: Electricity market price at time step *t* [$/MWh]
μ: Time resolution of electricity market [h]
*S* ^*ini*^: Initial hydrogen amount in storage [kg]
*C* ^*S*^: Hydrogen storage mass capacity [kg]
*C* ^*S*,*out*^: Output flow capacity of storage [kg/h]
$D_{i}^{min}$: Minimum hydrogen demand over subperiod *i* [kg]
$S_{k}=\left|S_{k}\right|$: Number of time slots assigned to electrolyzer *k*
$N_{k}=\left|N_{k}\right|$: Length of a single time slot assigned to electrolyzer *k*
**Scenario A**
λ^*su*^: Startup cost of the single electrolyzer [$]
*P* ^*max*^, *P* ^*min*^: Maximum and minimum power capacity of the single electrolyzer [MW]
*A*, *B*: Polynomial coefficients for the single electrolyzer [kg/MWh], [kg/h]
*R*: Ramping ratio of the single electrolyzer
**Scenarios B and C**
λ^*su*,*k*^: Startup cost of electrolyzer *k* [$]
*P* ^*max*,*k*^, *P* ^*min*,*k*^: Maximum and minimum power capacity of electrolyzer *k* [MW]
*A* ^*k*^, *B* ^*k*^: Polynomial coefficients for electrolyzer *k* [kg/MWh], [kg/h]
*R* ^*k*^: Ramping ratio of electrolyzer *k*
**Scenario C**
$P_{\tau }^{prot,k}$: Fixed power consumption of electrolyzer *k* at inner-slot time step τ, which is predefined via structured protocol [MW]

## Variables

$p_{t}^{e,sys}\in R^{+}$: Total power consumption of electrolyzer(s) in the system at time step *t* [MW]
$h_{t}^{e,sys}\in R^{+}$: Total hydrogen production of electrolyzer(s) in the system at time step *t* [kg/h]
$h_{t}^{d}\in R^{+}$: Hydrogen flow rate that is produced and immediately used on demand at time step *t* [kg/h]
$s_{t}\in R^{+}$: Amount of hydrogen currently stored at time step *t* [kg]
$s_{t}^{in},\;s_{t}^{out}\in R^{+}$: Hydrogen flow rate injected into, and released from the storage at time step *t* [kg/h]
$d_{t}\in R^{+}$: Hydrogen flow rate delivered to demand at time step *t* [kg/h]
**Scenario A**
$z_{t}^{on},\;z_{t}^{off}\in \left\{0,\;1\right\}$: Binary variable defining on and off state of the single electrolyzer at time step *t*
$z_{t}^{su}\in \left\{0,\;1\right\}$: Binary variable defining a cold startup of the single electrolyzer at time step *t*
**Scenarios B and C**
$p_{t}^{e,k}\in R^{+}$: Power consumption of electrolyzer *k* at time step *t* [MW]
$h_{t}^{e,k}\in R^{+}$: Hydrogen production of electrolyzer *k* at time step *t* [kg/h]
**Scenario B**
$z_{t}^{on,k},\;z_{t}^{off,k}\in \left\{0,\;1\right\}$: Binary variable defining on and off state of electrolyzer *k* at time step *t*
$z_{t}^{su,k}\in \left\{0,\;1\right\}$: Binary variable defining a cold startup of electrolyzer *k* at time step *t*
**Scenario C**
$p_{\sigma ,\tau }^{e,k}\in R^{+}$: Power consumption of electrolyzer *k* at time step τ in time slot σ [MW]
$h_{\sigma ,\tau }^{e,k}\in R^{+}$: Hydrogen production of electrolyzer *k* at time step τ in time slot σ [kg/h]
$u_{\sigma }^{on,k},\;u_{\sigma }^{off,k}\in \left\{0,\;1\right\}$: Binary variable defining on and off state of electrolyzer *k* in time slot σ
$u_{\sigma }^{su,k}\in \left\{0,\;1\right\}$: Binary variable defining a cold startup of electrolyzer *k* in time slot σ
$u_{\sigma }^{prot,k},\;u_{\sigma }^{max,k},\;u_{\sigma }^{min,k}\in \left\{0,\;1\right\}$: Binary variable defining the operating mode of electrolyzer *k* in time slot σ, whether to operate with the assigned protocol or at maximum or minimum power load
