# Supplementary material for: Achieving Flexible Operation by Aggregating Structured Operating Protocols: An Electrolysis Case Study Using Real Market Data
Source: Ind Eng Chem Res. 2026 Oct 2;65(40):21675–96. doi: 10.1021/acs.iecr.6c01628 (PMC13674073; doi:10.1021/acs.iecr.6c01628)
Supplement: Supplementary file 1 [file ie6c01628_si_001.pdf]

Supporting Information

Achieving Flexible Operation by Aggregating Structured Operating Protocols: An Electrolysis Case Study Using Real Market Data

Jukbin Kim[a], Pen-Yeh Yen[a], Abiela M. Bradley[b], Rachel N. Gaines[b]
Paul J. A. Kenis[b] and Victor M. Zavala[a*]

[a]Department of Chemical and Biological Engineering
University of Wisconsin-Madison, Madison, WI 53706, USA
[b]Department of Chemical and Biomolecular Engineering
University of Illinois Urbana-Champaign, Urbana, IL 61801, USA

*Corresponding Author: victor.zavala@wisc.edu.

S1 Economic Impact of Nonlinear Hydrogen Production

This section estimates the economic impact of using a nonlinear hydrogen production (HP) curve instead of its linearized approximation. Figure S1 compares the nonlinear HP data (red lines) and its linearized approximation (black dotted lines) in terms of the HP curve (left) and production efficiency curve (right). The data are regenerated using the simulation model developed in previous studies.[1,2] The linear model is constructed to match the data at the minimum and maximum power loads, following commonly used linearization approaches for electrolysis.[1,3] While industrial-scale analyses often adopt a linear production model with constant efficiency for computational simplicity, this approximation neglects the variation in production efficiency with power loading, as illustrated in the right panel of Figure S1. For structured protocols, such efficiency variation may have a notable impact on operational behavior and economic performance because the protocols are constrained to follow a series of predefined power loads. Thus, as a preliminary study prior to full optimization incorporating the nonlinear model, this section quantifies the potential economic impact of nonlinear HP through a posterior analysis of the operating profiles optimized with the linear model.

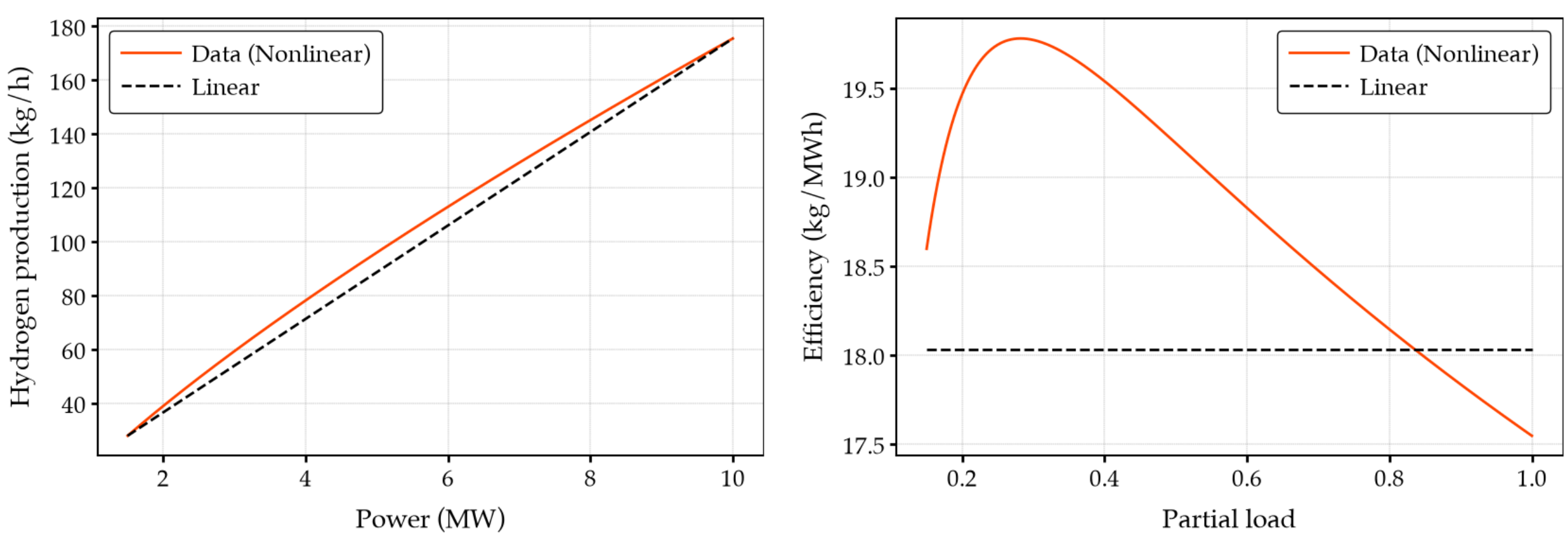

Figure S1: Hydrogen production (left) and efficiency (right) curves for a 10-MW alkaline electrolyzer. Red lines represent data obtained from hydrogen production models reported in previous studies,[1,2] which exhibit nonlinear behavior, while black dotted lines indicate the linearized model used in this study.

S1.1 Posterior calculation method

The post-optimization HP under the nonlinear model is estimated by replacing the constant efficiency, A $(= A^k)$, used in the linear models in Eqs. (6), (13), and (21) with a load-dependent efficiency function, $\eta(\hat{p})$. Here, $\hat{p}$ denotes the partial load, defined as the ratio of power consumption to the maximum power capacity. Based on the nonlinear efficiency data shown in the right panel of Figure S1, $\eta(\hat{p})$ is approximated by the fifth-order polynomial in Eq. (S1), with $R^2 = 0.9986$.

The optimized power consumption profiles are then retained and the corresponding hydrogen production is recalculated using $\eta(\hat{p})$. Because the power consumption profiles are fixed after optimization, the total electricity cost remains unchanged, while the amount of hydrogen produced

varies due to the load-dependent efficiency. The resulting change in hydrogen production is therefore reflected in the normalized electricity cost, allowing the economic impact of the nonlinear production model to be gauged without re-optimizing the operating profiles.

$$\eta(\hat{p}) = 92.551\hat{p}^5 - 303.11\hat{p}^4 + 386.06\hat{p}^3 - 237.91\hat{p}^2 + 67.146\hat{p} + 12.868 \quad \text{(S1)}$$

S1.2 Result and discussion

Figure S2 summarizes the normalized electricity costs in $\$/kgH_2$ for the different operational cases after posterior analysis. For each case, the bar consists of two components. The black-outlined portion represents the optimal normalized electricity cost obtained from the original optimization using the linear HP model, corresponding to the results in Figure 11. The red-outlined portion represents the subsequent cost adjustment obtained from the posterior calculation using the nonlinear HP model. A transparent red-outlined box indicates a cost increase, whereas a red-hatched box indicates a cost reduction. As in Figure 11, the economic performance of each operational case is compared against the baseline, with cost savings relative to the baseline reported as negative percentage values in parentheses.

The cost adjustments from the posterior analysis should be interpreted based on the operating profiles of individual electrolyzers rather than the aggregate power profiles, because the production efficiency depends on the partial load of each device. Specifically, the adjustments for scenarios (A), (B), and (C) can be interpreted together with the top panel of Figure 4, Figure 5, and Figures 8 – 10, respectively. Across these cases, individual electrolyzers tend to alternate between the minimum and maximum partial loads, where the nonlinear model exhibits lower production efficiency than at intermediate partial loads, as shown in the right panel of Figure S1. Relative to the the constant efficiency assumed in the linear HP model, the nonlinear efficiency is higher at the minimum partial load but lower at the maximum partial load. Because most operational cases spend a more fraction of time at the maximum power load, the lower efficiency at this load dominates, resulting in an increase in normalized electricity cost after the posterior calculation. In contrast, the baseline exhibits a cost reduction because it continuously operates at a partial load of approximately 0.75, where the nonlinear model predicts a higher efficiency. Despite these adjustments, the overall economic comparison among scenarios (A), (B), and (C) remains largely unchanged. In particular, the aggregation of structured protocols continues to approximate fully flexible operation: the scenario (C) cases achieve cost savings of 26.3% – 26.8% relative to the baseline, comparable to the 27.1% savings achieved by the $R = 1.0$ case of scenario (B).

One notable difference from Figure 11 is the reversal in the relative economic performance of cases with different ramping ratios within scenarios (A) and (B). After the posterior calculation, cases with the lowest ramping ratio ($R = 0.2$) exhibit greater cost savings than cases with higher ramping ratios. A lower ramping ratio restricts rapid transitions between the minimum and maximum loads, causing the electrolyzers to operate more frequently at intermediate-load regions where the nonlinear HP model predicts higher production efficiency. Consequently, the economic disadvantage

associated with reduced ramping flexibility is partially offset by the higher production efficiency at these intermediate loads. This result, however, does not demonstrate that lower-ramping operation is inherently more economical under a nonlinear HP model, because the nonlinear efficiency relationship is incorporated only through the posterior calculation and does not influence the original optimization decisions. Rather, the reversal between the original and posterior results highlights the importance of incorporating a more accurate HP model directly into the optimization framework. Such an extension would enable a consistent comparison of cases with different ramping capabilities while simultaneously accounting for electricity-price responsiveness and load-dependent efficiency variation.

The posterior analysis also provides preliminary insight into the design of structured protocols. As summarized in Table S1, the six cases in scenario (C) exhibit different cost adjustments depending on the power levels permitted within each case. Cases (a), which are restricted to the minimum and maximum power loads, consistently show costs increase of approximately 1.36%. In contrast, cases (b), which additionally allow operation at the protocol-specific intermediate power levels, show a smaller increase of approximately 1.21%, even lower than that observed for the fully flexible case. This result suggests that incorporating intermediate power levels into structured protocols can improve economic performance by exploiting regions of higher production efficiency. However, these intermediate operating levels may also be associated with higher degradation rates.[4,5] Therefore, selecting intermediate power levels based solely on production efficiency may compromise the durability benefits sought through structured operation. These insights suggest that structured-protocol specification should ultimately account for both load-dependent efficiency and degradation, highlighting an additional trade-off between economic efficiency and durability in protocol design.

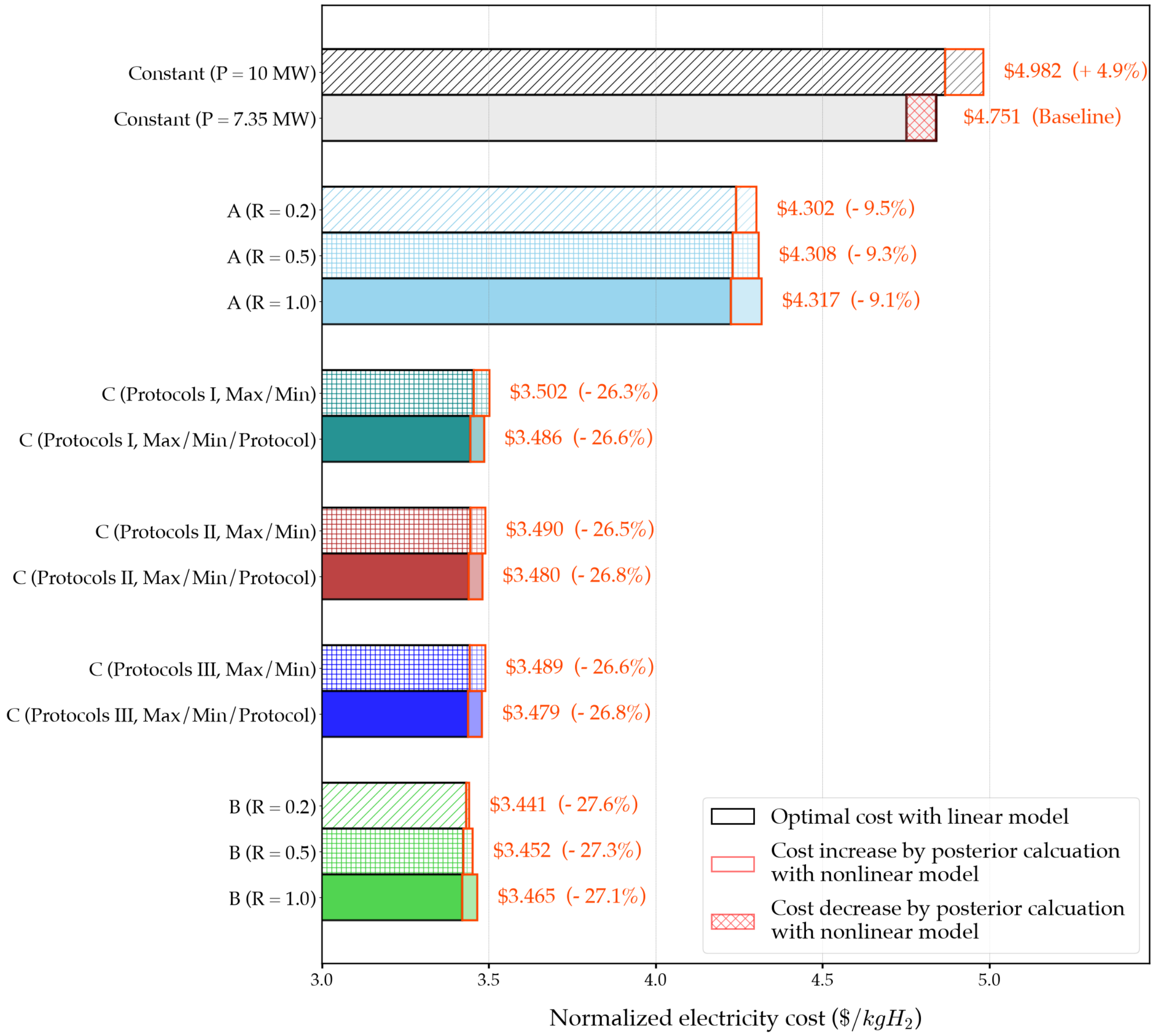

Figure S2: Summary of normalized electricity costs after the posterior analysis using the nonlinear HP model for all cases addressed in Figures 4 and 7. Black- and red-outlined portions represent the original optimized costs and their adjustments from the posterior analysis, respectively. Negative percentage values indicate cost savings relative to the baseline.

Table S1: Normalized electricity costs of scenario (C) and the fully flexible case in scenario (B) after the posterior analysis, as extracted from Figure S2, with percentage increases relative to the original optimal costs reported in Figure 11. Cases (a) and (b) correspond to two-mode (Max/Min) and three-mode (Max/Min/Protocol) operation, respectively.

Normalized electricity cost ($/kg)	**Protocols I**		**Protocols II**		**Protocols III**		scenario (B) ($R = 1.0$, reference)
	(a)	(b)	(a)	(b)	(a)	(b)	
Optimization with **linear** HP model	3.454	3.444	3.443	3.438	3.443	3.437	3.420
Posterior calculation with **nonlinear** HP model	3.502	3.486	3.490	3.480	3.489	3.479	3.465
$\Delta(\%)$	+1.37	+1.21	+1.35	+1.21	+1.35	+1.21	+1.33

References

(1) Raheli, E.; Werner, Y.; Kazempour, J. A conic model for electrolyzer scheduling. *Comput. Chem. Eng.* **2023**, *179*, 108450.

(2) Baumhof, M. T.; Raheli, E.; Johnsen, A. G.; Kazempour, J. Optimization of Hybrid Power Plants: When Is a Detailed Electrolyzer Model Necessary? 2023 IEEE Belgrade PowerTech. 2023.

(3) Valera, C.; Mostafa, M.; Zondervan, E. Modeling alkaline water electrolysis for power-to-x applications: A scheduling approach. *Int. J. Hydrogen Energy* **2021**, *46*, 9303–9313.

(4) Chandesris, M.; Médeau, V.; Guillet, N.; Chelghoum, S.; Thoby, D.; Fouda-Onana, F. Membrane degradation in PEM water electrolyzer: Numerical modeling and experimental evidence of the influence of temperature and current density. *Int. J. Hydrogen Energy* **2015**, *40*, 1353–1366.

(5) Dörner, S.; Kinzl, M.; Mitsos, A.; Bongartz, D. Dynamic lumped cell-level model of chemical membrane degradation in PEM electrolysis: Impact of pressure and time dependence. *Ind. Eng. Chem. Res.* **2025**, *64*, 24982–24993.
